# Morpho-Phonetic Effects in Speech Production: Modeling the Acoustic Duration of English Derived Words With Linear Discriminative Learning

**DOI:** 10.3389/fpsyg.2021.678712

**Published:** 2021-08-02

**Authors:** Simon David Stein, Ingo Plag

**Affiliations:** English Language and Linguistics, Department of English and American Studies, Heinrich Heine University Düsseldorf, Düsseldorf, Germany

**Keywords:** speech production, linear discriminative learning, acoustic duration, morphological theory, derivation, mental lexicon

## Abstract

Recent evidence for the influence of morphological structure on the phonetic output goes unexplained by established models of speech production and by theories of the morphology-phonology interaction. Linear discriminative learning (LDL) is a recent computational approach in which such effects can be expected. We predict the acoustic duration of 4,530 English derivative tokens with the morphological functions DIS, NESS, LESS, ATION, and IZE in natural speech data by using predictors derived from a linear discriminative learning network. We find that the network is accurate in learning speech production and comprehension, and that the measures derived from it are successful in predicting duration. For example, words are lengthened when the semantic support of the word's predicted articulatory path is stronger. Importantly, differences between morphological categories emerge naturally from the network, even when no morphological information is provided. The results imply that morphological effects on duration can be explained without postulating theoretical units like the morpheme, and they provide further evidence that LDL is a promising alternative for modeling speech production.

## Introduction

Recent findings in morpho-phonetic and psycholinguistic research have indicated that phonetic detail can vary by morphological structure. For example, the acoustic duration of English word-final [s] and [z] differs depending on morphological status and inflectional function (Plag et al., 2017, 2020; Seyfarth et al., 2017; Tomaschek et al., 2019). For derivation, too, studies have demonstrated effects of morphological structure on phonetic output. For example, morphological geminates in English differ in duration depending on morphological category and informativity (Ben Hedia and Plag, 2017; Ben Hedia, 2019), and phonetic reduction in various domains can depend on how easily speakers can decompose a complex word into its constituents (e.g., Hay, 2003, 2007; Plag and Ben Hedia, 2018; Zuraw et al., 2020).

These findings raise several problems at the theoretical level. The observation that phonetic detail varies systematically with morphological properties is unaccounted for by traditional and current models of the morphology-phonology interaction and of speech production (e.g., Chomsky and Halle, 1968; Kiparsky, 1982; Dell, 1986; Levelt et al., 1999; Roelofs and Ferreira, 2019; Turk and Shattuck-Hufnagel, 2020). This is because these models are either underspecified regarding the processing of complex words, or do not allow for post-lexical access of morphological information. For example, feed-forward models of the morphology-phonology interface (e.g., Kiparsky, 1982) assume that morphological brackets around constituents are “erased” in the process of passing on a word through morphological and phonological levels of processing. This means that no trace of morphological structure should be left at the level of phonetic realization. Similarly, established psycholinguistic models of speech production (e.g., Levelt et al., 1999) assume that morphological units select general phoneme templates which are then passed on to an articulator module to be realized phonetically. Again, no morphological information is encoded in these templates, meaning that no systematic differences between morphological properties are expected at the phonetic level.

Yet, morphological effects on the phonetic output have repeatedly been observed, which is incompatible with these assumptions. For example, the observation that complex words are more acoustically reduced when they are less decomposable into their constituents (Hay, 2003, 2007; Plag and Ben Hedia, 2018; Zuraw et al., 2020) seems to suggest that information about morphological boundaries must somehow still be present at the phonetic level. From the perspective of the speech production models and theories of the morphology-phonology interaction outlined above, such effects are unexpected, and the mechanisms behind them are unclear. To better explain the morphology-phonetics interaction at the theoretical level and to understand the patterning of durations in complex words from a new perspective, we need alternative approaches.

One such approach is to model phonetic detail based on the principles of discriminative learning (see, e.g., Ramscar and Yarlett, 2007; Ramscar et al., 2010; Baayen et al., 2011). Such an approach sees form-meaning relations not as compositional, but as discriminatory instead. That is, form-meaning relations are created in a system of *difference*, which distinguishes between features based on their similarity and dissimilarity and connects them to each other in a learning process. In discriminative approaches, “signs” in the semiotic sense of relations of form and meaning (de Saussure, 1916) are not fixed units. Discriminative models refrain from sub-lexical static representations such as morphemes or roots in the lexicon. Instead, speech comprehension and production are the result of a dynamic learning process where relations between form and meaning are constantly recalibrated based on the speaker's experience. How strong associations between given forms and meanings are in the system depends on how often specific forms occur together with specific meanings, and on how often they fail to occur together with others. Each time a speaker makes a new experience, i.e., encounters a form together with a specific meaning, all associations of forms and meanings in the system are updated to reflect this new state of learning. An association strength increases when a “cue” (such as a specific form) occurs together with an “outcome” (such as a specific meaning), and an association strength decreases when a cue does not occur with the outcome.

Such an approach has clear advantages if we are to explain the evidence that morphology directly affects phonetic realization. A discriminative learning model lacks a feed-forward architecture which divides speech processing into separate levels. It is an end-to-end model that goes directly from form to meaning and from meaning to form. This means that the loss of morphological information between levels, e.g., through bracket erasure or phoneme template selection, is no longer an issue. Moreover, discriminative learning refrains from postulating morphemes or phonemes as psychologically relevant units in the first place. This opens the way for interpreting acoustic differences from a new perspective. In a discriminative approach, differences between morphological functions are expected to emerge naturally from sublexical and contextual cues. If we can model systematic acoustic variation between morphological functions with measures derived from a discriminative network, it is possible to explain potential effects by its theoretical principles of learning and experience.

While discriminative approaches have already been used to model other morphological correlates, such as reaction time (e.g., Baayen et al., 2011), the question arises whether a discriminative approach is able to successfully predict phonetic variation. Recently, Tomaschek et al. (2019) employed naïve discriminative learning (NDL) to model the duration of English word-final [s] and [z] of different morphological status. The measures derived from their network were predictive and indicated that a higher certainty in producing a morphological function leads to lengthening. While Tomaschek et al. (2019) focused on inflection, it is necessary to also test how well discriminative approaches can deal with derivational morphology. The present paper aims to account for this gap.

Our study investigates the durational properties of derived words in English. We modeled word durations for 4,530 tokens with the derivational functions dis, ness, less, ation, and ize from the Audio BNC (Coleman et al., 2012), using multiple linear regression models and mixed-effects regression models. The crucial predictors in our models are measures derived from the computational framework of linear discriminative learning (Baayen et al., 2019b).

Linear discriminative learning (LDL) is a new variant of naïve discriminative learning. Like NDL, it is *discriminative* because its system of form-meaning relations is generated by discriminating between different forms and meanings instead of building them from compositional units. Like NDL, LDL is a system of *learning* because the association strengths between forms and meanings are continuously recalibrated in a process of experience. This learning is simple and interpretable because, in contrast to deep learning, it features just two layers, an input layer and an output layer, both of which are linguistically transparent. Unlike NDL, however, LDL is *linear* and no longer “naïve.” Its networks are linear mappings between form matrices and meaning matrices (which serve as either the input layer or the output layer, respectively). In this approach, forms are represented by vectors, and meanings are also represented by vectors, similarly to approaches in distributional semantics. The idea is that if we can express both forms and meanings numerically, we can mathematically connect form and meaning. In LDL, the network is no longer naïve because where NDL represents word meanings with binary vectors, LDL uses real-valued vectors, taking into account that words cannot only be similar in form, but also in meaning. How this is implemented is explained further below in the section Materials and Methods.

Our aim in this study is, first, to investigate how well LDL can account for the durational variation in our data. Second, we investigate what the effects of the LDL-derived measures tell us about the mechanisms of speech production. How can we interpret potential effects conceptually? Third, as we are interested in exploring how these findings relate to morphological functions, we also investigate how the results differ depending on how much information the network has about these functions. For this purpose, we initially trained three different LDL networks, two of which contain explicit morphological information. The first network does not include any information about morphological category and treats all derivatives as idiosyncratic (the Idiosyncratic Network). The second network uses vectors that include semantic information about the derivative and about the morphological category it belongs to (the Morphology Network). The third network uses vectors that include semantic information about the base word (instead of the derivative) and about the morphological category (the Base Network).

We hypothesize that LDL-derived measures can successfully (i.e., significantly) predict derivative durations. If they do, the effects of LDL-derived measures should be interpretable with regards to speech production (for example, they should mirror the finding by Tomaschek et al. (2019) that higher certainty is associated with longer durations). Lastly, we explore whether there are differences between the networks that contain information about the morphological category a derivative belongs to and the network that does not contain such information.

To preview our results, three key findings emerge from the analysis. First, all LDL networks achieve high learning accuracy and the proportion of variance in duration explained by the LDL-derived predictors is comparable to that explained by traditional predictors. Second, the effects of LDL measures highlight important patterns of speech production. For example, they suggest that words are lengthened in speech production when the semantic support of the word's predicted articulatory path is stronger (i.e., when certainty is higher), mirroring the finding by Tomaschek et al. (2019). Third, we find that, even though we did not provide the Idiosyncratic Network with any information about the morphological category a word belongs to, these categories still emerge from the network. For instance, the different morphological categories are reflected in the distributions of the correlation strength of a word's predicted semantics with the semantics of its neighbors. This corresponds to what we would traditionally describe as the differences in semantic transparency between affix categories.

The remainder of this paper is structured as follows. The section Materials and Methods describes our methodology, illustrating the procedure of collecting the speech data (the section Speech Data), building the LDL networks (the section Linear Discriminative Learning), the variables used (the section Variables) and the modeling procedure (the section Modeling Word Durations). The section Results outlines our results, followed by a discussion and conclusion in the section Discussion and Conclusion.

## Materials and Methods

Our methodology consists of three main steps: first, retrieving the speech data for the durational measurements for the response variable, second, building the LDL networks to retrieve LDL-derived predictors of interest, and third, devising regression models to predict derivative durations from various predictors. All data, scripts and materials can be found at osf.io/jkncb.

### Speech Data

The speech data was obtained from the Audio BNC (Coleman et al., 2012). This corpus consists of both monologues and dialogues from different speech genres of several British English varieties. It comes phonetically aligned by an automatic forced aligner. Containing about 7.5 million words, it is large enough to yield enough observations per derivational function. A corpus approach has the advantage that that we are not only able to analyze a lot of data, but also that the type of data is conversational speech. This enables us to investigate a more authentic process of language production than with carefully elicited speech. It has been argued (e.g., Tucker and Ernestus, 2016) that research on speech production in particular needs to shift its focus to spontaneous speech to be able to draw valid conclusions about language processing.

The morphological categories selected for investigation are dis, ness, less, ation, and ize. We use the term *morphological category* in the traditional sense, referring to words that share a particular morphologically expressed meaning. We do not use the term *morpheme* because it is usually employed to denote a minimal sign combining a form and a meaning (e.g., /-ləs/ “without,” see, e.g., Plag and Balling, 2020). We use the term *function* to refer to the semantic or grammatical contribution of a particular affix or process. LDL does not assume any fixed relationship between form and meaning. Meanings are dynamically mapped onto a stream of forms (overlapping triphones in our case), but never defined as being tied to strings that we would traditionally describe as being “morphemic.” The terms *function* and *category* better reflect the fact that in LDL, derived words might be grouped into categories sharing similar semantics or features (cf. Word and Paradigm Morphology) but are not “composed” of form-meaning building blocks (cf. morpheme-based morphology). LDL's lexomes are pointers to meanings only, not to forms.

The five categories dis, ness, less, ation, and ize were chosen, first, because they featured sufficient token counts in the Audio BNC and are attested in Baayen et al.'s (2019b) vector space (explained in the section Training Data). Second, they were chosen because they cover a wide spectrum of characteristics traditionally considered important for affix classification. For example, following Bauer et al. (2013) and Plag (2018), the affixes corresponding to those categories differ in their semantic transparency: *-ness, -less*, and *dis-* produce mostly transparent derivatives, whereas *-ize* and *-ation* are overall a little less transparent in comparison. They vary in the range of their meanings, from relatively narrow and clearly definable semantics (e.g., the privative meaning of *-less* or the negative meaning of *dis-*) to more varied semantics (e.g., *-ness* denoting abstract states, traits, or properties) to highly multifaceted semantics (*-ize* can have locative, ornative, causative, resultative, inchoative, performative, or similative meaning, *-ation* can denote events, states, locations, products or means). They also differ in their productivity, with *-ness* and *-less* being considered highly productive, and *-ize, -ation*, and *dis-* being somewhat less productive. Lastly, they also differ phonologically. While *-ness, -less*, and *dis-* are not (obligatorily) subject to phonological alternations and not involved in resyllabification processes, *-ize* and *-ation* can cause stress shifts and other phonological alternations within their bases, and resyllabification is commonplace.

We obtained speech data for these morphological categories by entering pertinent query strings into the web interface of the Audio BNC and extracting the resulting wordlist and associated recordings and textgrids. These query strings searched for all word tokens that begin or end in the orthographic and phonological representation of each of the investigated derivational function. We manually cleaned the datasets by excluding words which were monomorphemic (e.g., *bless, disk, station*), whose semantics or base were unclear (e.g., *harness, disrupt, dissertation*), or which were proper names or titles (e.g., *Guinness, Stenness, Stromness*).

Before starting the acoustic analysis, manual inspection of all items was necessary to exclude items that were not suitable for further analysis. This was done by visually and acoustically inspecting the items in the speech analysis software Praat (Boersma and Weenik, 2001). Items were excluded that fulfilled one or more of the following criteria: the textgrid was a duplicate or corrupted for technical reasons, the target word was not spoken or was inaudible due to background noise, the target word was interrupted by other acoustic material, laughing, or pauses, the target word was sung instead of spoken, the target word was not properly segmented or incorrectly aligned to the recording. In cases where the alignment did not seem satisfactory, we examined the word-initial boundary and the word-final boundary in order to decide whether to exclude the item. We considered an observation to be correctly aligned if none of these boundaries would have to be shifted to the left or right under application of the segmentation criteria in the pertinent phonetic literature (cf. Machač and Skarnitzl, 2009; Ladefoged and Johnson, 2011). Following Machač and Skarnitzl (2009), we considered the shape of the sound wave to be the most important cue, followed by the spectrogram, followed by listening.

In a final step, the dataset was reduced to only those words that were attested in the TASA corpus as well as in CELEX, and whose base was simplex (this step is explained in the section Training Data). The final dataset of derivatives that entered the models comprised 4,530 tokens and 363 types. Table 1 gives an overview of the data in each morphological category. Further descriptive statistics of the datasets are provided in the Supplementary Material.

**Table 1:** Overview of tokens and types per morphological category.

	**DIS**	**NESS**	**LESS**	**ATION**	**IZE**
Tokens	233	344	145	3,403	405
Types	35	49	31	209	39

### Linear Discriminative Learning

Our aim is to predict the durational patterning in the 4,530-token dataset described above with measures derived from an LDL network. These measures can be calculated on the basis of a transformation matrix that maps a cue matrix *C* for forms onto a semantic matrix *S* for meanings (for comprehension), and the semantic matrix *S* onto the cue matrix *C* (for production). The basic building blocks used to construct the meaning dimensions in matrix *S* are referred to as *lexomes*. Lexomes are atomic units of meaning in an LDL network and serve as pointers to semantic vectors. In comprehension, they are also the “outcomes” in the *S* matrix, which are predicted from the “cues” in the *C* matrix. Lexomes can for example correspond to words (content lexomes, such as lemon), but also to derivational or inflectional functions (function lexomes, such as ness).

It is important to note that function lexomes correspond to morphological categories, but are not the same thing as morphemes. In LDL, morphological categories (like ness) are coded as semantic vectors and are not units of form and meaning, but units of meaning only. How these lexomes and their vectors were obtained, how the matrices were constructed and how they were mapped onto each other is illustrated in the following sections.

#### Training Data

To construct a linear discriminative learning network, it is necessary to obtain semantic vectors that represent the words' meanings (this will be explained in more detail in the section Matrices for Form and Meaning). For this, we made use of the vectors generated by Baayen et al. (2019b) from the TASA corpus, who used an algorithm to predict words in each sentence of the corpus from other words in that sentence (this will be explained further below). To make sure that we can use these semantic vectors for our derivatives, we first reduced our speech data set from the Audio BNC to those derivatives that are attested in TASA (losing 352 words). In a second step, we used the CELEX lexical database (Baayen et al., 1995) to obtain phonological transcriptions for the words in our data set. These transcriptions are necessary for constructing the matrices. Since CELEX did not have transcriptions for all words, this step led to a slight reduction of our data set (losing 9 words). In a final step, we excluded all derivatives (49 words) whose bases were already complex, i.e., all derivatives that have more than one derivational function (e.g., *stabilization, specification, attractiveness, disclosure, disagreement*). One reason for excluding these derivatives is that it is currently not clear how to build their semantic vectors. Another reason is that multi-affixed words in corpora are comparatively infrequent. Too infrequent derivatives might require a corpus even bigger than TASA from which to construct reliable semantic vectors.

The resulting dataset contained 363 unique derivatives (i.e., types). This dataset consists of all derivatives from the Audio BNC that are also attested in TASA. One problem with this dataset is that it would be rather unrealistic as training data. This is because a speaker encounters far more than just a few hundred words during their lifetime, and not all these encountered words contain one of the five investigated morphological categories dis, ness, less, ation, and ize. We therefore decided to merge this dataset with all words in TASA that had already been coded in Baayen et al. (2019b) for derivational functions (function lexomes) and phonological transcriptions (4,880 more words). This dataset contained 897 derivatives with the 25 derivational function lexomes again, agent, dis, ee, ence, ful, ic, instrument, ation, ish, ist, ive, ize, less, ly, ment, mis, ness, not, ordinal, ous, out, sub, undo, and y, as well as 3,983 monomorphemic words. Derivational functions were coded irrespective of variation in affix spelling. Most of these words are not attested in our speech data and therefore not of interest for the durational modeling, but including them makes the training itself more realistic.

The resulting 5,176 unique word forms were then used for the *C* matrix, and the 5,201 unique lexomes (comprising the vectors for the 5,176 content lexomes and the 25 derivational function lexomes) were used for the *S* matrix. The next section illustrates what these matrices are and how they are constructed.

#### Matrices for Form and Meaning

In an LDL network, features of a word are represented by a vector for this word in a multidimensional space. Each word has a vector that specifies its form features, and a vector that specifies its semantic features. We therefore need two matrices: a cue matrix *C* for the words' forms and a semantic matrix *S* for the words' meanings.

The cue matrix *C* contains in rows the words' phonological transcriptions, and in columns form indicators that are either present or absent in those words. As shown in Arnold et al. (2017) and Shafaei-Bajestan et al. (2020), it is possible to use real-valued features extracted directly from the speech signal instead of discrete features. In the present study, we use triphones as form indicators, following Baayen et al. (2019b). These triphones overlap and can be understood as proxies for transitions in the articulatory signal. Each cell in the matrix codes in a binary fashion (1 for present or 0 for absent) whether the respective triphone string (specified in the column) occurs in the phonological transcription of the word (specified in the row). An example of the layout of the *C* matrix is given in Table 2 on the left-hand side. For the *C* matrix in this study, we used the 5,176 unique word forms mentioned in the section Training Data.

**Table 2:** Schematic examples of a cue matrix *C* (left) and a semantic matrix *S* (right) for the words *cat, happiness, walk*, and *lemon*.

**Schematic example of a** ***C*** **matrix**						**Schematic example of an** ***S*** **matrix**				
	**#k{**	**k{ t**	**{t#**	**#h{**	**h{p**		**CAT**	**HAPPINESS**	**WALK**	**LEMON**
k{t	1	1	1	0	0	k{t	0.000000	**−**6.24e-05	4.71e-05	**−**0.000138
h{pInIs	0	0	0	1	1	h{pInIs	**−**0.000110	0.0000000	0.000194	**−**2.20E-05
w$k	0	0	0	0	0	w$k	0.000304	**−**0.0002335	0.000000	**−**3.74E-05
lEm@n	0	0	0	0	0	lEm@n	**−**7.28e-05	**−**2.41e-07	**−**2.68e-05	0.00000

*Note that for the triphones in the C matrix, word boundaries are also counted, represented by a hash (#). The DISC phonetic alphabet is used for computer-readable transcription (Burnage, 1990)*.

The semantic matrix *S* contains in its rows the words' phonological transcriptions, and in its columns the semantic dimensions, or lexomes, with which the words are associated. In the present study, these lexomes correspond to interpretable linguistic items, such as words and derivational functions. Each cell in the S matrix contains a real number, which represents the association strength of a word (specified in the row) to a lexome (specified in the column). As mentioned in the Introduction, this is an important difference of LDL compared to NDL, where word meanings are initially coded as binary-valued vectors similar to the cue matrix. LDL, on the other hand, starts out with real-valued association weights. An example of the layout of the *S* matrix is given in Table 2 on the right-hand side. For the *S* matrix in this study, we used the 5,201 unique lexomes mentioned in the section Training Data.

Where do these association weights come from? In the present study, we used association weights that were generated from word co-occurrence in real language data. For this, Baayen et al. (2019b) trained an NDL network on the TASA corpus (Ivens and Koslin, 1991; Landauer et al., 1998). This NDL network operated on an established learning algorithm (Widrow and Hoff, 1960) that incrementally learns association strengths between lexomes. In such an approach, words in a sentence are predicted from the words in that sentence. While the network goes through the sentences in the corpus, the associations strengths of the lexomes with each other are continuously adjusted over time. As language learning is about learning which connections are relevant, the association strength of lexomes that often occur together will be strengthened. As discriminative learning is also about *un*learning connections which are irrelevant, similarly, the association strength of lexomes will be weakened each time they do not occur together. For the implementational and mathematical details of this procedure, as well as for the validation of the resulting semantic vector space, the reader is referred to Baayen et al. (2019b). Importantly for the present study, Baayen and colleagues included lexomes not only for words, but also for derivational functions corresponding to suffixes and prefixes. This enables us to build LDL networks that take into account morphological categories shared between derivatives (in addition to an LDL network that does not take these into account and treats all words as idiosyncratic, i.e., as having a unique semantics that is not related to the semantics of constituents below the word level).

The so-called *lexome-to-lexome matrix* resulting from this learning process is a vector space in which each lexome vector represents a certain association with the meanings of all other lexomes. According to the idea that “you shall know a word by the company it keeps” (Firth, 1957), each value in the vector of a lexome represents the association strength of this lexome to the meaning of another lexome in TASA. Following Baayen et al. (2019b), we used a version of their lexome-to-lexome matrix which was trimmed to about five thousand dimensions and whose main diagonal was set to zero.^1^ From this lexome-to-lexome matrix, we extracted the vectors for our 5,201 unique lexomes (described in the section Training Data), which we then used for the *S* matrix.

For the present study, we built three different LDL networks: one which contains no information about the morphological category a derivative belongs to but treats all derivatives as idiosyncratic, one in which the vectors contain information about the derivative and about the morphological category it belongs to, and one in which the vectors contain information about the base of a derivative and about the morphological category it belongs to. For each of these networks we need a matrix *S* and a matrix *C*. We will refer to the matrices with idiosyncratic derivatives as matrix *S*_*I*_ and matrix *C*_*I*_, to the matrices with information about the derivative and its morphological category as matrix *S*_*M*_ and matrix *C*_*M*_, and to the matrices with information about the base and the morphological category as matrix *S*_*B*_ and matrix *C*_*B*_. We will refer to the networks as a whole as the *Idiosyncratic Network*, the *Morphology Network*, and the *Base Network*, respectively.

The Idiosyncratic Network with matrices *S*_*I*_ and *C*_*I*_ considered only the semantic vector of the derivative lexome (e.g., only the vector for happiness, which can be represented as $\vec{happiness}$). This vector was taken as is from the lexome-to-lexome matrix and straightforwardly entered matrix *S*_*I*_ for each word. This way, the vector contains only idiosyncratic information, and no information about any shared morphological category.

The Morphology Network with matrices *S*_*M*_ and *C*_*M*_ made use of the semantic vector of the content lexome of the derivative (e.g., the vector for happiness, i.e., $\vec{happiness}$) and the semantic vector of the corresponding derivational function lexome (e.g., the vector for ness, which can be represented as $\vec{NESS}$).^2^ We took both these vectors from the lexome-to-lexome matrix, and the sum of these two vectors entered matrix *S*_*M*_ for each word. That is, the semantic vector associated with the word *happiness* was the sum of the vectors for happiness and ness: $\vec{happiness}+\vec{NESS}$. This way, the resulting vector contains idiosyncratic information, but also information about the morphological category it shares with other derivatives. While it is also conceivable to add to the vector of ness the vector of happy (instead of happiness), taking happiness better reflects the fact that derived words most often still carry some idiosyncratic meaning, i.e., signify more than merely the sum of their parts. The combination of happiness and ness, thus, takes into account the morphological category ness that the word shares with other derivatives, but still acknowledges that English derivatives are not characterized by strictly compositional semantics.

The Base Network with matrices *S*_*B*_ and *C*_*B*_ uses the semantic vectors of the content lexomes of the bases of derived words and the vectors of the derivational function lexomes. That is, instead of adding the derivational lexome vector to the lexome vector of the derivative as in the Morphology Network, in the Base Network we add the derivational lexome vector to the content lexome vector of the derivative's base. For instance, the semantic vector associated with the word *happiness* in matrix *S*_*B*_ is the sum of the vectors for happy and ness: $\vec{happy}+\vec{NESS}$. This way, the resulting vector contains information about the morphological category it shares with other derivatives, like in the Morphology Network. But unlike the Morphology Network, it contains no idiosyncratic information at all. The meaning of complex words in the Base Network is assumed (against our better knowledge) to be strictly compositional. In principle, this property makes this network unattractive and less suitable for predicting word durations, but it can be fruitfully used to gain further insights into the differences between architectures.

We now have three matrices (for each morphological setup, respectively) of the layout shown in Table 2. We have the *C* matrix, containing information about form, and the *S* matrix, containing information about meaning. These matrices can now be mapped onto each other.

#### Comprehension and Production Mapping

In speech comprehension, a listener encounters a form and needs to arrive at the corresponding meaning. Therefore, for comprehension we calculate a transformation matrix *F* which maps the semantic matrix *S* onto the cue matrix *C*, so that

(1) $$\begin{array}{r} CF=S. \end{array}$$

In speech production, on the other hand, a speaker starts out with a meaning and needs to find the right form to express this meaning. Therefore, for production we calculate a transformation matrix *G* which maps the cue matrix *C* onto the semantic matrix *S*, so that

(2) $$\begin{array}{r} SG=C. \end{array}$$

**Figure 1:**
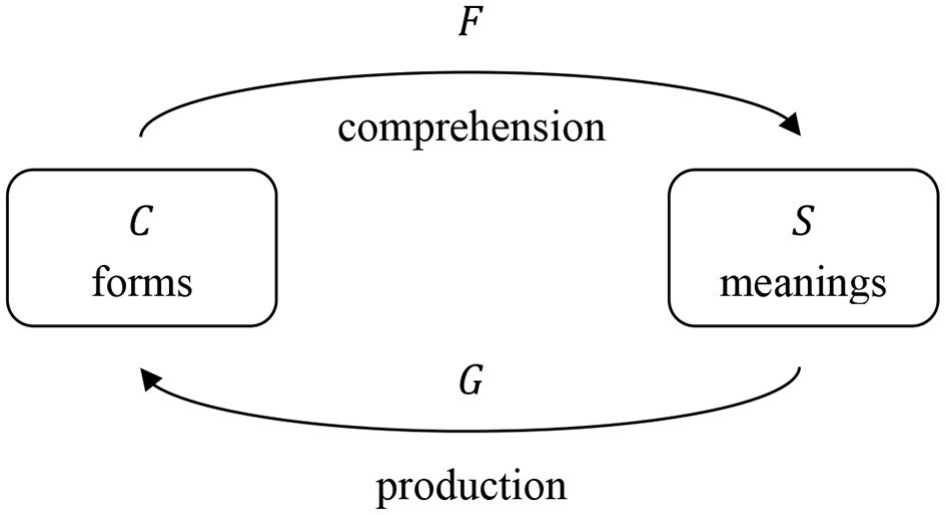
Comprehension and production mapping, adapted from Baayen et al. (2019b). For comprehension, transformation matrix *F* transforms the cue matrix *C* into the semantic matrix *S*. For production, transformation matrix *G* transforms the semantic matrix *S* into the cue matrix *C*.

Mathematically, the transformation matrices *F* and *G* can be calculated by multiplying the generalized inverse (Moore, 1920; Penrose, 1955) of *C* with *S* (for comprehension) and the generalized inverse of *S* with *C* (for production). The transformations are visually illustrated in Figure 1.

As soon as we have obtained the transformation matrices, we can use them to estimate what forms and meanings the network would predict. For this, we calculate the predicted matrices Ŝ and Ĉ. For comprehension, we multiply the form matrix *C* with the transformation matrix *F*, i.e., we solve Ŝ = *CF*. For production, we multiply the semantic matrix *S* with the transformation matrix *G*, i.e., we solve Ĉ = *SG*. It is important to keep in mind that the mappings are simple linear transformations that are achieved by matrix multiplication (for an introduction in the context of LDL, see Baayen et al., 2019b). It is possible to think of the transformation matrices *F* and *G* like coefficients in linear regression, which try to approximate the target matrix but will not produce exactly the same values. This is true especially for large datasets like in the present study. The predicted matrices Ŝ and Ĉ are thus not exactly the same as the original matrices *S* and *C*.

We can also use the predicted matrices to evaluate model accuracy. To see how well the model predicts the semantics of an individual word in comprehension, we can multiply an observed form vector *c* from the cue matrix with the transformation matrix *F* to obtain a predicted semantic vector ŝ. We can then see how similar this predicted semantic vector ŝ is to the target semantic vector *s*. For production, in turn, we can multiply an observed meaning vector *s* from the semantic matrix with the transformation matrix *G* to obtain the predicted form vector ĉ, which represents the estimated support for the triphones. We can then see how similar this predicted form vector ĉ is to the target form vector *c*. If the correlation between the estimated vector and the targeted vector, i.e., between ŝ and *s* or between ĉ and *c*, respectively, is the highest among the correlations, a meaning or form is correctly recognized or produced. The overall percentage of correctly recognized meanings or forms is referred to as comprehension accuracy and production accuracy, respectively.

To obtain the mappings, we used the learn_comprehension() and learn_production() functions from the R package WpmWithLDL (Baayen et al., 2019a). Accuracy estimations were obtained with the functions accuracy_comprehension() and accuracy_production(). Finally, the measures of interest which we use to predict the durations were extracted from the networks with the help of the comprehension_measures() function and the production_measures() function. While we model word durations in the present study, which are the result of speech production, both speech production and speech comprehension mappings produce relevant measures for the analysis of production data. This is because the emergent structure of the learner's lexicon is determined both by the association of forms with meanings and of meanings with forms. In LDL, like in human learning, production and comprehension are inextricably linked to each other (see Baayen et al., 2019b for discussion). We will now describe the LDL-derived measures, as well as other used measures, in more detail.

### Variables

As described above, many potentially useful LDL measures can be extracted automatically from the matrices by the package WpmWithLDL (Baayen et al., 2019a). However, some of the variables provided by this package capture similar things and are strongly correlated with each other. Careful variable selection, and sometimes adaptation, was therefore necessary. Further below we illustrate our selection and explain the conceptual dimensions we aim to capture with each variable.

Conceptually, it is desirable to not have any traditional linguistic covariates in the models that are not derived from the network, such as lexical frequencies, neighborhood densities, or bigram frequencies. It is important to build models instead which contain LDL-derived variables only. This is because, first, we are interested in how well an LDL network fares on its own in predicting speech production. Second, many traditional covariates bring along implicit assumptions that LDL does not want to make, such as the existence of discrete phonemic and morphemic units. Third, it is unclear how these traditional measures contribute to learning and processing. At the same time, however, the traditional measures might tap into properties of the linguistic signal that are picked up in a discriminative learning process. Hence, LDL measures often correlate with traditional measures.^3^

The models of interest therefore only include LDL-derived variables (described in the section LDL-Derived Predictor Variables), with one exception: the one important non-LDL variable that needs to be taken into account is speech rate. This is an influence that is beyond the control of the network.

In addition, we built models with just non-LDL variables (we describe these variables in the section Traditional Predictor Variables). This is to compare the explanatory power of the LDL-derived models with traditional models used in morpho-phonetic research.

#### Response Variable

##### Duration Difference

One important problem in analyzing spontaneous speech is that which words are spoken is uncontrolled for phonological and segmental makeup. This problem is particularly pertinent for the present study, as our datasets feature different affixes whose derivatives vary in word length. To mitigate potential durational differences that arise simply because of the number and type of segments in each word, we refrained from using absolute observed duration as our response variable. Instead, we derived our duration measurement in the following way.

First, we measured the absolute acoustic duration of the word in milliseconds from the textgrid files with the help of scripts written in Python. Second, we calculated the mean duration of each segment in a large corpus (Walsh et al., 2013) and computed for each word the sum of the mean durations of its segments.^4^ This sum of the mean segment durations is also known as “baseline duration,” a measure which has been successfully used as a covariate in other corpus-based studies (e.g., Gahl et al., 2012; Caselli et al., 2016; Sóskuthy and Hay, 2017; Engemann and Plag, 2021). It would now be possible to subtract this baseline duration from the observed duration, giving us a new variable that represents only the difference in duration to what is expected based on segmental makeup. However, we found that this difference is not constant across longer and shorter words. Instead, the longer the word is on average, the smaller the difference between the baseline duration and the observed duration. In a third and final step, we therefore fitted a simple linear regression model predicting observed duration as a function of baseline duration. The residuals of this model represent our response variable. Using this method, we factor in the non-constant relationship between baseline duration and observed duration. We named this response variable duration difference, as it encodes the difference between the observed duration and a duration that is expected on the basis of the segmental makeup.

#### LDL-Derived Predictor Variables

##### Mean word SUPPORT

**Figure 2:**
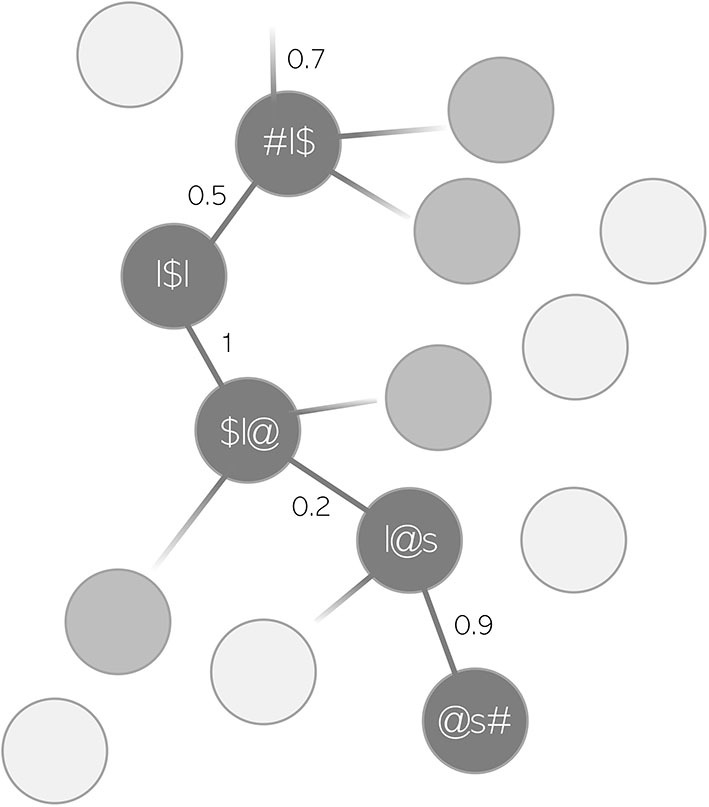
Toy example of an articulatory path for the word *lawless*. Each connection between a triphone node is assigned a probability of being selected against other triphones.

mean word support is a measure that we introduce to capture how well-supported on average transitions from one triphone to the next are in the production of a word. Taken together, these transitions are referred to as an articulatory “path.” mean word support is calculated based on the variable path sum from the package WpmWithLDL. path sum refers to the summed semantic support that a given predicted articulatory path receives from its corresponding predicted semantic vector ŝ, i.e., the path from one triphone to the next in the predicted form of a word. This is illustrated in Figure 2 with the toy example *lawless*. Each node in the path, i.e., each triphone, has a certain probability of being selected against all the other possible triphones when trying to produce a word based on its semantics. The maximum value per transition is therefore 1, i.e., a 100% probability of being selected. However, with longer words, there are also more transitions. For example, if a word's form is perfectly predicted across all triphone transitions, but there are five such transitions, path sum would take the value 5. Thus, the problem with path sum is that it increases not only with higher support, but also with increasing segmental length of words. This would not be ideal as a measure of semantic support when modeling durations, since durations naturally increase with longer words. The interpretation of path sum as a measure for mere semantic support would be difficult. Therefore, we decided to divide each value of path sum, i.e., each summed support of a word's path, by the number of path nodes in a word. This new variable mean word support controls for path length and only reflects the average transition support in each word. mean word support can be read as a metaphor for certainty. The higher the average transition probabilities in a word, the more certain the speaker is in pronouncing this word based on its semantics. Based on previous studies which have found higher certainty of various operationalizations to be associated with lengthening (Kuperman et al., 2007; Cohen, 2014, 2015; Tomaschek et al., 2019; Tucker et al., 2019), words with higher mean word support can be expected to be longer in duration.

##### Path Entropies

Like mean word support, path entropies considers the transition probabilities between nodes in the path from one triphone to the next in the predicted form of a word. path entropies is the Shannon entropy calculated over the support that a given path in the predicted form vector ĉ receives from its corresponding predicted semantic vector ŝ. Entropy is a measure of the uncertainty in the choice of one of several alternatives. Higher entropy generally means a larger number of possibilities of similar probabilities, in other words, less certainty. Similarly to mean word support, this measure is thus related to certainty, albeit in a conceptually different way. The higher the entropy, the less certain the speaker is in producing a word, because there is not much informational value in the path support differences. Higher path entropies thus indicate more uncertainty. Based on the above-mentioned previous studies on certainty (Kuperman et al., 2007; Cohen, 2014, 2015; Tomaschek et al., 2019; Tucker et al., 2019), words with higher path entropies can thus be expected to be shorter.

##### Semantic Vector Length

semantic vector length refers to the L1 distance, also known as taxicab distance, Manhattan distance, or city-block distance, of ŝ. It thus measures the length of the predicted semantic vector by summing the vector's absolute values. We decided to use the L1 distance instead of the correlated L2 distance, as the former does not lose information by smoothing over the city-block distance. The longer the predicted semantic vector becomes, the stronger the links to other lexomes become. semantic vector length can thus be understood as a measure of semantic activation diversity. It is the extent to which a given word predicts other words. As a result, it can also be understood as a measure of polysemy. The more semantic dimensions a speaker is active on for a word and the more other meanings the word can predict, the more collocational relations it has and the more varied and confusable the meanings of this word are (cf. Tucker et al., 2019, also cf. the notion of “sense uncertainty” in Filipović Durdević and Kostić, 2021). Following Tucker et al. (2019), words with higher activation diversity can be expected to be shorter: the speaker is more uncertain when more meanings are activated and therefore invests less energy in maintaining the signal.

##### Semantic Density

semantic density refers to the mean correlation of ŝ with the semantic vectors of its top 8 neighbors' semantic vectors. A strong average correlation of the estimated semantic vector with the vectors of its neighbors means that the neighboring words are semantically very similar to the word in question. The higher the density, the more semantically similar these words are. semantic density applied to derived words is thus an important measure of semantic transparency: Words in a dissimilar neighborhood are idiosyncratic and their meaning is not predictable. Words in a semantically similar neighborhood are semantically transparent, i.e., mathematically shifted in the same direction. It is currently unclear whether one should expect a facilitatory or inhibitory effect of measures related to semantic transparency on duration. We explore this question in more detail in the discussion in the section Discussion and Conclusion.

##### Target Correlation

target correlation refers to the correlation between a word's predicted semantic vector ŝ and the word's target semantic vector *s*. This is a measure for how accurate the network is in predicting meaning based on form. The closer the predicted meaning to the actual targeted meaning, the more successful is the model, and the better is the learner in making the correct connection between form and meaning. Being better in making the correct connection between form and meaning could be expected to have a facilitatory effect in both comprehension and production, i.e., in our case, to lead to shorter durations.

#### Traditional Predictor Variables

##### Speech Rate

speech rate is the only covariate in our LDL-derived models, and the only predictor that is not derived from the LDL networks. It is, of course, also used in the traditional models. The duration of a word is naturally influenced by how fast we speak. speech rate can be operationalized as the number of syllables a speaker produces in a given time interval (see, e.g., Pluymaekers et al., 2005b; Plag et al., 2017). In the window containing the target word plus 1 s before and 1 s after it, we divided the number of syllables by the duration of this window. This is a good compromise between a maximally local speech rate which just includes the adjacent segments, but allows the target item to have much influence, and a maximally global speech rate, which includes larger stretches of speech but is vulnerable to changing speech rates during this larger window. The number of syllables in the window and the duration of this window were extracted from the textgrids with a Python script. A higher speech rate (i.e., more syllables being produced within the window) should lead to shortening.

##### Word Frequency

word frequency has been shown to affect acoustic durations (and processing in general) in many different studies (for an overview, see, e.g., Baayen et al., 2016). Higher word frequency is expected to lead to shorter durations. We extracted the word frequency, i.e. the frequency of the derivative, from the *Corpus of Contemporary American English* (COCA, Davies, 2008), with the help of the corpus tool Coquery (Kunter, 2016). Derived words are often rare words (see, e.g., Plag et al., 1999). For this reason, very large corpora are necessary to obtain frequency values for derived words. We chose COCA because this corpus is much larger than the BNC, and therefore had a much higher chance of the words and their bases being sufficiently attested. We prioritized covering a bigger frequency range with more tokens. Following standard procedures, we log-transformed word frequency before it entered the models instead of using raw frequency. We added a constant of +1 to the variable in order to be able to take the log of the zero frequency of non-attested derivatives (cf. Howes and Solomon, 1951; Baayen, 2008).

##### Relative Frequency

relative frequency refers to the frequency of the base word relative to the frequency of its derivative from COCA (Davies, 2008), calculated by dividing base frequency by word frequency. It is a frequency-based measure for morphological decomposability. Morphological decomposability, or segmentability, has been found to affect duration in a number of studies (Hay, 2003, 2007; Pluymaekers et al., 2005b; Schuppler et al., 2012; Zimmerer et al., 2014; Ben Hedia and Plag, 2017; Plag and Ben Hedia, 2018; Zuraw et al., 2020). The higher the relative frequency, the more decomposable the item is assumed to be. According to Hay (2001, 2003, 2007), more decomposable words should feature longer durations (although some studies have also found the opposite). We added a constant of +1 and log-transformed the variable.

##### Bigram frequency

bigram frequency refers to the frequency of the target derivative occurring together with the word following it in the COCA (Davies, 2008). It has been found that the degree of acoustic reduction can be influenced by the predictability of the following context (see, e.g., Pluymaekers et al., 2005a; Bell et al., 2009; Torreira and Ernestus, 2009). It is thus expected that the higher the bigram frequency, the shorter the duration. We added a constant of +1 and log-transformed the variable.

##### Mean Biphone Probability

The variable biphone probability refers to the sum of all biphone probabilities (the likelihood of two phonemes occurring together in English) in a given target derivative. It has been found that segments are more likely to be reduced or deleted when they are highly probable given their context (see, e.g., Munson, 2001; Edwards et al., 2004; Turnbull, 2018; also see Hay, 2007 on transition legality effects on reduction). Thus, biphone probability can be expected to negatively correlate with duration: the more probable the biphones, the shorter the durations. Biphone probabilities were calculated by the Phonotactic Probability Calculator (Vitevitch and Luce, 2004). For this, we first manually translated the target derivatives' ASCII transcriptions of the Audio BNC into the coding referred to as Klattese, as this is the computer-readable transcription convention required by this calculator.

##### AFFIX

affix is a categorical variable coding which affix category the derivative belongs to. This is to account for any potential idiosyncrasies in durations of affix categories.

### Modeling Word Durations

Due to the distributional properties of the words in our dataset, we decided to fit both standard multiple linear regression models and mixed-effects regression models to the data. In our dataset, we have many types that are attested only once, which precludes the use of mixed-effects regression.^5^ Having many single observations for one type involves the danger that certain word types may become too influential in the model. Mixed-effects regression, on the other hand, can prevent certain word types from being too influential in the model but necessitates the exclusion of items for which no repeated measurements are available. We decided to address this problem by fitting and documenting both types of model. All regression models were fitted in R (R Core Team, 2020), using the lme4 package (Bates et al., 2015) and lmerTest (Kuznetsova et al., 2016) for the mixed models.

In the course of fitting the regression models, we trimmed the dataset by removing observations from the models whose residuals were more than 2.5 standard deviations away from the mean, which led to a satisfactory distribution of the residuals (see, e.g., Baayen and Milin, 2010). For the standard regression models, this resulted in a loss of 82 observations (1.8% of the data) for the model based on the Idiosyncratic Network, and 74 observations (1.6% of the data) for the models based on the Morphology Network and the Base Network.

For the mixed models, we only included word types that occurred more than once (reducing our dataset from 363 to 261 types, or from 4,530 to 4,358 observations). The trimming procedure resulted in a loss of 71 observations (1.6% of the data) for the models based on the Idiosyncratic Network and the Base Network, and 70 observations (1.6% of the data) for the model based on the Morphology Network.

From our experience, LDL-derived variables are often strongly correlated with each other. As explained in the section Variables, we made sure to select variables that are not highly correlated and that had least conceptual overlap with each other, in terms of representing specific concepts such as certainty or semantic transparency. Still, we used variance inflation factors to test for possible multicollinearity of the remaining variables. All of the VIF values were smaller than 2, i.e., far below the critical value of 10 (Chatterjee and Hadi, 2006).

The initial models were fitted including all variables described in the section Variables. The models were then simplified according to the standard procedure of removing non-significant terms in a stepwise fashion. An interaction term or a covariate was eligible for elimination when it was non-significant at the 0.05 alpha level. Non-significant terms with the highest *p*-value were eliminated first, followed by terms with the next-highest *p*-value. This was repeated until only variables remained in the models that reached significance at the 0.05 alpha level.

## Results

### General Comparison of the Networks

Network accuracy was generally satisfactory, with comprehension accuracies at 81, 82, and 83% for the Idiosyncratic Network, the Morphology Network, and the Base Network, respectively, and production accuracies at 99, 99, and 98%, respectively.

Before turning to the regression models that predict duration, let us compare the predicted semantic matrices Ŝ of the three networks. This can be done by calculating the correlation of each predicted semantic vector ŝ from one network with its corresponding predicted semantic vector ŝ from the other two networks, and then taking the mean of these correlations for all words. Comparing the semantic vectors $\hat{s_{I}}$ of the Idiosyncratic Network to the semantic vectors $\hat{s_{M}}$ from the Morphology Network, we find that they are on average very weakly correlated: the mean correlation between the vectors of the $\hat{S_{I}}$ matrix and the $\hat{S_{M}}$ matrix was *r* = 0.08. This means that the matrices are rather different. Likewise, the mean correlation between the vectors of the $\hat{S_{I}}$ matrix and the $\hat{S_{B}}$ matrix is weak (*r* = 0.1).

However, the mean correlation between the vectors of the $\hat{S_{M}}$ matrix and the $\hat{S_{B}}$ matrix is extremely high (*r* = 0.9). This indicates that it is probably the information about derivational function that differentiates the semantic vectors of the Idiosyncratic Network from the semantic vectors of the other two networks. Morphological category matters.

### Predicting Durations With LDL Variables

Let us now turn to the regression models predicting duration. Tables 3, 4 report the final models regressing duration difference against the LDL-derived variables and speech rate.

**Table 3:** Final standard linear regression models reporting effects on duration difference with variables from the three networks.

	**Idiosyncratic Network model**			**Morphology Network model**			**Base Network model**		
	**Estimate**	**SE**		**Estimate**	**SE**		**Estimate**	**SE**	
Intercept	0.216901	0.026210	^***^	0.090708	0.025887	^***^	0.408246	0.029999	^***^
MEAN WORD SUPPORT	0.170726	0.023507	^***^	0.250262	0.020700	^***^	0.050723	0.012716	^***^
PATH ENTROPIES	−0.008688	0.002242	^***^	−0.008442	0.002309	^***^	−0.009342	0.002259	^***^
SEMANTIC DENSITY	−0.043545	0.008925	^***^	0.033868	0.012372	^**^	−0.093906	0.025844	^***^
SPEECH RATE	−0.058757	0.001148	^***^	−0.058602	0.001159	^***^	−0.058702	0.001171	^***^
*N*	4,448			4,456			4,456		
*R* ^2^ *adjusted*	0.3778			0.3742			0.3623		

*Full models are documented in the Supplementary Material. Significance codes:*

*** *< 0.001*,

** *< 0.01*,

* *< 0.05*.

**Table 4:** Final mixed-effects regression models reporting effects on duration difference with variables from the three networks.

	**Idiosyncratic Network model**			**Morphology Network model**			**Base Network model**		
	**Estimate**	**SE**		**Estimate**	**SE**		**Estimate**	**SE**	
Intercept	1.328e-01	4.601e-02	^**^	2.146e-01	6.024e-02	^***^	2.595e-01	2.510e-02	^***^
MEAN WORD SUPPORT	2.722e-01	4.600e-02	^***^	2.535e-01	4.572e-02	^***^	1.211e-01	2.654e-02	^***^
PATH ENTROPIES	−1.173e-02	5.625e-03	*	−1.163e-02	5.633e-03	*			
SEMANTIC VECTOR LENGTH	−1.606e-02	6.860e-03	*	−3.294e-02	1.550e-02	*			
SPEECH RATE	−5.944e-02	1.116e-03	^***^	−5.937e-02	1.116e-03	^***^	−5.936e-02	1.117e-03	^***^
*N*	4,357			4,358			4,357		
*R* ^2^ *marginal*	0.3690016			0.3638608			0.3487138		
*R* ^2^ *conditional*	0.5198377			0.5168201			0.5200542		

*Full models are documented in the Supplementary Material. Significance codes:*

*** *< 0.001*,

** *< 0.01*,

* *< 0.05*.

The model in Table 3 reports the results of the standard regression models. As we can see, of the LDL-derived variables, mean word support, semantic density, and path entropies significantly affect duration in the regression models of all three networks. In addition, speech rate is significant in all three models. The variables semantic vector length and target correlation, on the other hand, did not reach significance and were therefore excluded from these final models.

The model in Table 4 reports the results of the mixed models. These models are very similar to the standard regression models, with two important differences. The variables mean word support and speech rate display the same effects as in the standard models. path entropies also displays the same effects for the Idiosyncratic Network and the Morphology Network (it was only marginally significant for the Base Network and therefore excluded). However, semantic density does not reach significance in the mixed models. Instead, there is a significant effect of semantic vector length in the models derived from the Idiosyncratic Network and the Morphology Network, but not in the Base Network.

Before taking a look at the effects of individual variables, let us first examine how much variation is actually explained by the models. Tables 3, 4 show that for all three networks in both types of model, the *R*^2^ of the fixed effects is between 0.36 and 0.37, i.e., about 36–37% of the variance in duration is explained by the predictors (the marginal *R*^2^ of the mixed model for the Base Network is an exception, being slightly lower with about 35%). To put this number into perspective, we compared the explained variance of the LDL-derived models to that of a model containing predictor variables that are traditionally used in morpho-phonetic corpus studies of duration. We fitted a standard linear regression model and a mixed model including the traditional predictors from the section Traditional Predictor Variables. These variables were fitted to the response variable duration difference. Some observations were lost due to the same trimming procedure as explained in the section Modeling Word Durations (80 observations, or 1.8% of the data, for the standard model, and 74 observations, or 1.7% of the data, for the mixed model). For the sake of comparison of the explanatory power of individual predictors, we did not remove insignificant variables from the models. The models are summarized in Table 5. word frequency, relative frequency, and bigram frequency were not significant in the models, while mean biphone probability, some levels of affix, and speech rate were. We can see that about the same proportion of the variance is explained by the traditional models (*R*^2^ = 0.37).

**Table 5:** Standard linear regression model and mixed-effects regression model reporting effects on duration difference with traditional, non-LDL predictors.

	**Traditional standard regression model**			**Traditional mixed-effects model**		
	**Estimate**	**SE**		**Estimate**	**SE**	
Intercept	3.888e-01	8.345e-03	^***^	4.159e-01	1.106e-02	^***^
WORD FREQUENCY	4.970e-08	3.764e-08		−2.608e-07	2.328e-07	
RELATIVE FREQUENCY	−2.136e-05	4.166e-05		−1.446e-05	8.931e-05	
BIGRAM FREQUENCY	−6.542e-07	6.293e-07		7.978e-07	6.382e-07	
MEAN BIPHONE PROBABILITY	−5.188e+00	8.872e-01	^***^	−7.167e+00	1.545e+00	^***^
AFFIX ATION						
DIS	8.145e-03	6.700e-03		−1.405e-03	1.438e-02	
IZE	−2.316e-02	5.251e-03	^***^	−1.491e-02	1.377e-02	
LESS	−5.749e-02	8.226e-03	^***^	−7.569e-02	1.524e-02	^***^
NESS	−5.473e-02	5.700e-03	^***^	−3.630e-02	1.295e-02	^**^
SPEECH RATE	−5.893e-02	1.163e-03	^***^	−5.986e-02	1.116e-03	^***^
*N*	4,450			4,354		
*R* ^2^ *adjusted/marginal*	0.3731			0.3705799		
*R* ^2^ *conditional*				0.5344904		

*Full models and ANOVAs are documented in the Supplementary Material. Significance codes:*

*** *< 0.001*,

** *< 0.01*,

* *< 0.05*.

Partitioning how much each of the predictors contributes to the proportion of explained variance, using the lmg metric (Lindeman et al., 1980) from the relaimpo package (Grömping, 2006) and the calc.relip.mm function (Beresewicz, 2015) reveals that in both the traditional models and the LDL models, by far most of the variance is explained by speech rate (which alone explains about 35% of the total variance in the standard regression models and about 20% in the mixed models). This is shown in Table 6. The variables of interest mean word support, path entropies, semantic density, and semantic vector length are all comparable in their explanatory power to the categorical affix variable and mean biphone probability, and often better than the three frequency measures word frequency, relative frequency, and bigram frequency. While the small differences in the explained variance between the LDL-derived variables and the traditional variables after factoring out the contribution of speech rate are not large enough to truly say which set of variables is “better,” they clearly show that they are in the same ballpark. We can thus say that LDL-derived variables can compete against traditional variables from morpho-phonetic studies.

**Table 6:** Relative importance of variables in the models for the overall explained variance (marginal variance for mixed models).

	**Relative importance metrics (lmg)**							
	**Idiosyncratic**		**Morphology**		**Base**		**Traditional**	
	**Network**		**Network**		**Network**		**model**	
	***lm***	***lmer***	***lm***	***lmer***	***lm***	***lmer***	***lm***	***lmer***
MEAN WORD SUPPORT	0.0089	0.1649	0.0148	0.0956	0.0025	0.1641		
PATH ENTROPIES	0.0023	0.0031	0.0023	0.0017	0.0030			
SEMANTIC DENSITY	0.0067		0.0020		0.0014			
SEMANTIC VECTOR LENGTH		0.0064		0.0399				
SPEECH RATE	0.3605	0.1946	0.3556	0.2266	0.3559	0.1845	0.3561	0.2140
WORD FREQUENCY							0.0007	0.0065
RELATIVE FREQUENCY							0.0006	0.0044
BIGRAM FREQUENCY							0.0007	0.0034
MEAN BIPHONE PROBABILITY							0.0025	0.1178
AFFIX							0.0136	0.0246
Total variance explained	0.3778	0.3690	0.3742	0.3639	0.3623	0.3487	0.3731	0.3706

**Figure 3:**
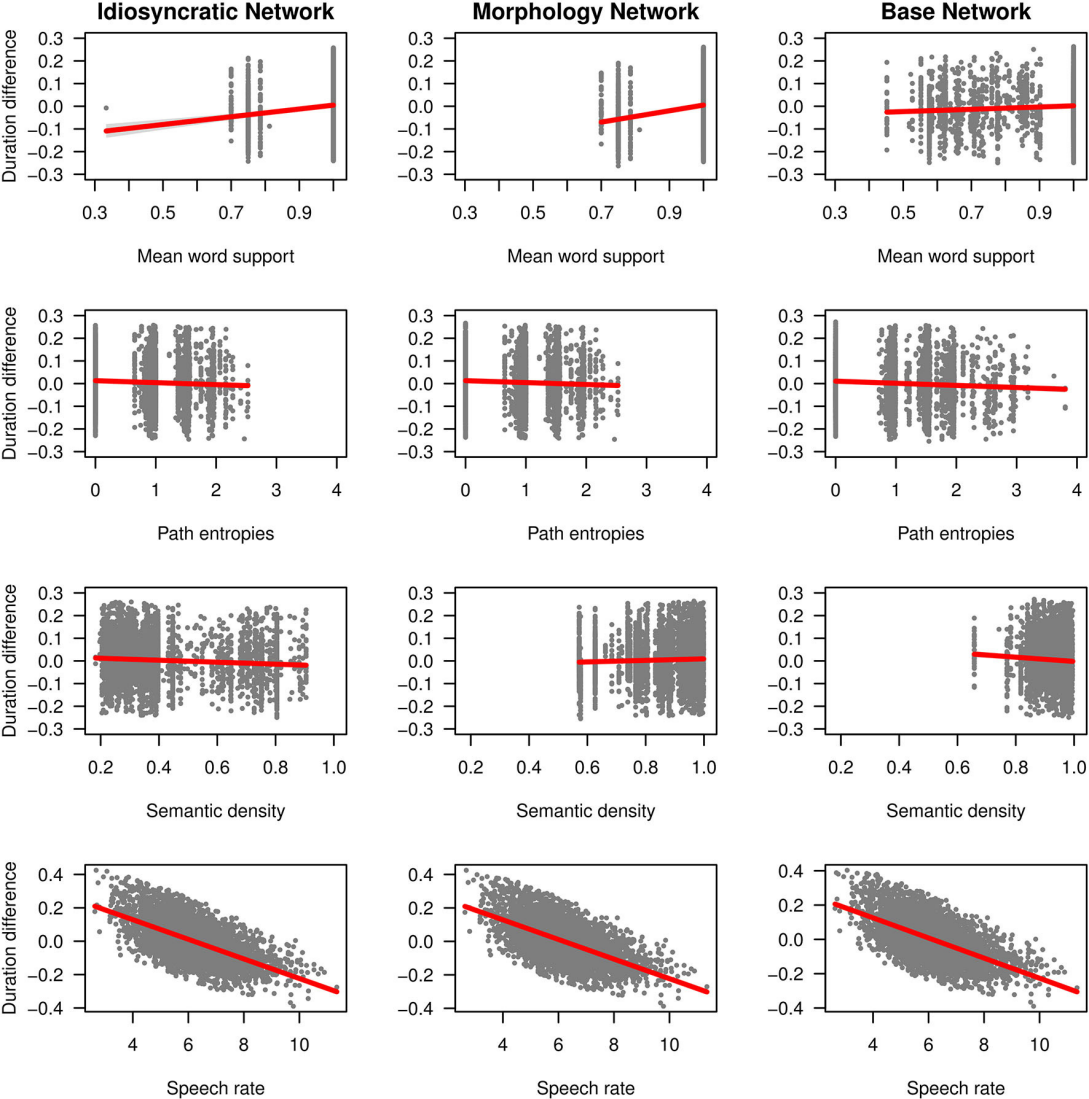
Effects on duration difference in the standard linear regression models for the Idiosyncratic Network variables (left column), the Morphology Network variables (middle column) and the Base Network variables (right column).

**Figure 4:**
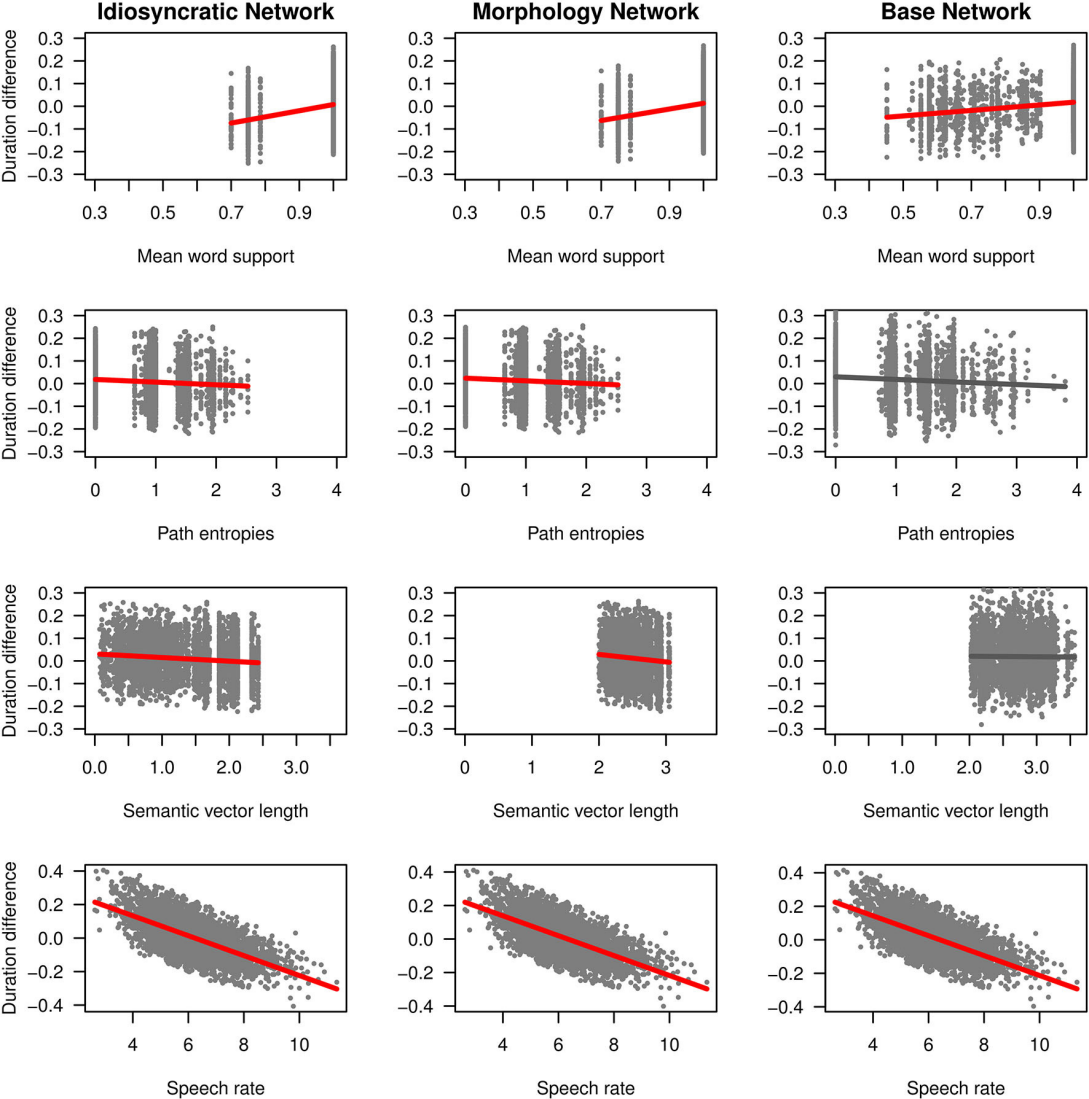
Effects on duration difference in the mixed-effects regression models for the Idiosyncratic Network variables (left column), the Morphology Network variables (middle column) and the Base Network variables (right column). Red regression lines indicate significant effects from the final models, gray regression lines indicate non-significant effects from the initial models before the non-significant predictors were excluded.

**Figure 5:**
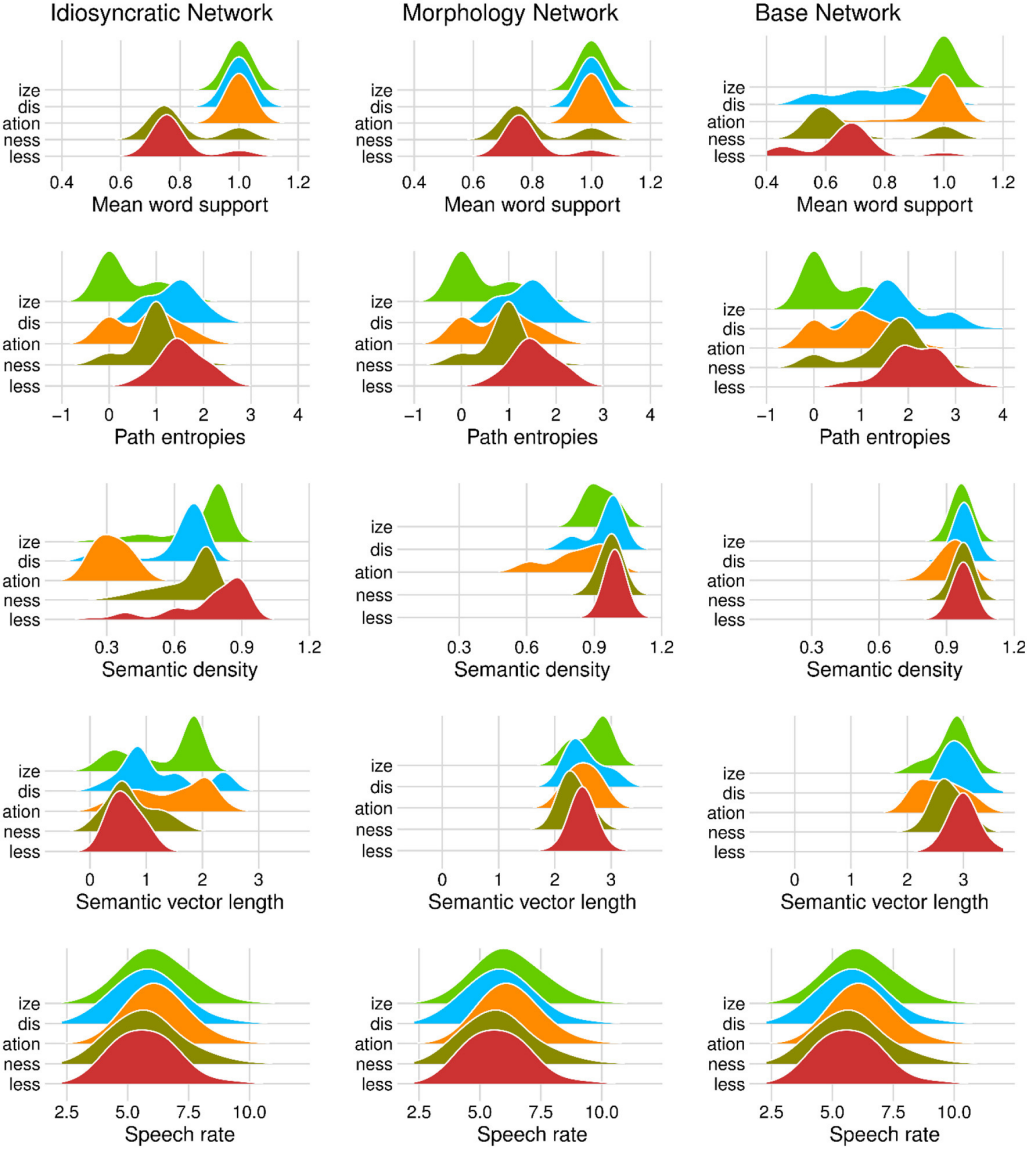
Density distributions of variables by derivational function in the Idiosyncratic Network models (left column), the Morphology Network models (middle column), and the Base Network models (right column). Note that in the first two panels in the top tow, the density curves around 1.0 are calculated over a single value.

We can now take a closer look at the effects of each of the variables. Figure 3 (for the standard regression models) and Figure 4 (for the mixed models) plot the effects of the LDL-derived variables and speech rate on duration. Figure 5 displays the density distributions of the variables in all three networks. We will discuss the two variables relating to certainty in the articulatory path first (mean word support and path entropies), followed by a discussion of the two variables relating to the semantic relations between words (semantic density and semantic vector length). The covariate speech rate and the variable target correlation will not be further discussed, as speech rate behaves as expected (see the bottom rows of Figures 3, 4) and target correlation was not significant in any of the models.

#### Mean Word Support and Path Entropies

As explained in the section LDL-Derived Predictor Variables, the two variables mean word support and path entropies both reflect properties of the semantic support for the predicted articulatory path, and they both tap into articulatory certainty. Given that the way these variables are calculated, mean word support is a measure of certainty, while path entropies is a measure of uncertainty, they should mirror each other by showing opposite effects on duration. We find that this is the case.

Let us start with mean word support. This variable has a significant effect on duration difference in all models. We can see from the coefficients in Tables 3, 4 as well as from its positive slope in the top row of Figures 3, 4 that higher mean word support is significantly associated with longer durations. The higher the average semantic support of a word's predicted triphone path, the longer this word is pronounced. This means that the more certain the speaker is in producing the word, the more the articulation is durationally enhanced. In other words, more certainty is associated with lengthening. Interestingly, if we look at the distribution of mean word support in the top row of Figure 5, we can see that mainly two derivational functions are responsible for this effect: Whereas the paths of ize and ation words are always very well-supported (as well as the paths of dis in the Idiosyncratic Network and in the Morphology Network), paths of ness and less words often feature weaker transition probabilities between triphones. The distributional differences of each of these two categories compared to the others are significant (Mann-Whitney, *p* < 0.001). This is true for all three networks. However, it is notable that the mean support of words is generally lower in the Base Network, especially for ize, ness, and less words. We will come back to these differences between morphological categories and between networks in the discussion.

If mean word support indicates that with greater certainty, durations become longer, our next predictor path entropies should indicate that with greater uncertainty, durations become shorter. This is the case. Moving on to the second row in Figures 3, 4, we can observe negative slopes for the effect of path entropies, which was significant in the models (marginally significant in the mixed model for the Base Network). The higher the Shannon entropy of the semantic support for the predicted articulatory paths becomes, i.e., the more variation of support there is in the system, the shorter the durations are. More uncertainty is associated with reduction. In other words, a speaker's lower certainty in production means the articulatory signal is less strengthened or less enhanced. Again, there are differences between morphological categories in all three networks. For example, words with ize are characterized by more diverse and informative support values, while the other categories often feature more entropic supports across the paths, especially less and dis. All differences in the distributions are significant at *p* < 0.001, except for the non-significant difference between less and dis in the Idiosyncratic Network and the Morphology Network, and the difference between ness and dis in the Base Network.

#### Semantic Density and Semantic Vector Length

Let us now look at the two variables that capture the semantic relations to other words, semantic density and semantic vector length.

semantic density is significant in the standard regression models, but did not reach significance in the mixed models. Its coefficients in Table 3 show that while it has a negative effect on duration when derived from the Idiosyncratic Network and the Base Network, it has a positive effect on duration when derived from the Morphology Network. This is illustrated in the third row of Figure 3. For the Idiosyncratic Network and the Base Network, the stronger an estimated semantic vector correlates with its neighbors, the shorter becomes the duration of a word. For the Morphology Network, the stronger an estimated semantic vector correlates with the semantic vectors of its neighbors, the longer becomes the duration of a word. High-density words live in a space more semantically close to other words, i.e., they can be said to be less idiosyncratic and, due to their being derived words, more semantically transparent. Higher transparency can thus lead to both lengthening and shortening, depending on how the network is constructed.

Investigating the distribution of this variable, we observe that semantic density shows differences between the networks. The data points in Figure 3 and the distributions in Figure 4 show that density is lowest in the Idiosyncratic Network, higher in the Morphology Network, and highest in the Base Network. This means that density increases with the amount of morphological structure we encode in the networks. semantic density also shows differences between derivational functions. Especially in the Idiosyncratic Network, this difference is very pronounced. This is again illustrated in Figure 5 (third row, first column). Words with less and ize have particularly high densities, whereas densities are lower for dis and ness words, and lowest for ation words. All of the distributions are significantly different from each other at *p* < 0.001. The fact that these morphological categories cluster so distinctly is particularly surprising, given that the Idiosyncratic Network was not provided with any information about these categories. We will return to the peculiar behavior of this variable in the discussion.

Turning to the second semantic variable, we can see that semantic density is replaced by semantic vector length in the mixed models: semantic vector length, while not significant in the standard regression models, reaches significance in the mixed models for the Idiosyncratic Network and the Morphology Network (Table 4 and third row in Figure 4). When derived from these networks, semantic vector length has a negative effect on duration. Recalling that this variable captures activation diversity, we can say that being active on more semantic dimensions as a speaker has a facilitatory effect in production. The more collocational relations a word has to other words and the more meanings are activated, the shorter it is pronounced.

Investigating the distribution of semantic vector length (Figure 5, fourth row), we observe that the estimated semantic vectors are generally longer in the Morphology Network and the Base Network than in the Idiosyncratic Network. Not only are they longer on average, they also cluster more closely together in terms of their length: the L1 distance in the Morphology Network and the Base Network covers a range from about 2 to 3, while in the Idiosyncratic Network, it is spread out across a range from about 0 to 2.5. One reason for this may be purely mathematical: The vectors in the two networks with information about the morphological category can often be longer because the vector for the derivational function lexome is added to the vector of the derived word's content lexome. However, the vectors are not just generally longer in these networks, but the spread of the datapoints is also narrower. This indicates that the words cluster more closely together. Since semantic vector length can represent activation diversity, this is expected: If words share a morphological function with other words, they become more similar to each other, hence are more likely to be semantically active when a member of their category is accessed. In the Idiosyncratic Network, words do not explicitly share a morphological category, hence members of a given category are not as likely to be co-activated. Again, the distributions show that vector lengths cluster differently depending on derivational function, meaning that different morphological categories are characterized by different degrees of semantic activation diversity.

It is interesting to note that when modeling durations, it is the Base Network that seems to behave differently from the other two networks, even though it shares with the Morphology Network its property of having information about morphological categories. The mixed model based on the variables from the Base Network is the least successful, as two predictors that are significant in the other networks (path entropies and semantic vector length) do not reach significance in the Base Network. In the section Matrices for Form and Meaning, we have already discussed that the Base Network is conceptually unappealing and theoretically flawed, as it wrongly assumes that the meaning of a derived word is strictly composed of the meaning of its base word and the meaning of the affix. However, we now find that it also seems to perform less optimal in modeling durations. Importantly, it is surprising that the Base Network shows a facilitatory effect of semantic density similar to the Idiosyncratic Network, instead of behaving like the Morphology Network, i.e., showing an inhibitory effect. This is despite the fact that the distribution of semantic density is very similar in the Base Network and in the Morphology Network, but very different in the Idiosyncratic Network (see again Figure 5, third row). Moreover, it was the $\hat{S_{M}}$ matrix and the $\hat{S_{B}}$ matrix which are extremely highly correlated with each other (see the section General Comparison of the Networks) and not at all correlated with the $\hat{S_{I}}$ matrix.

**Figure 6:**
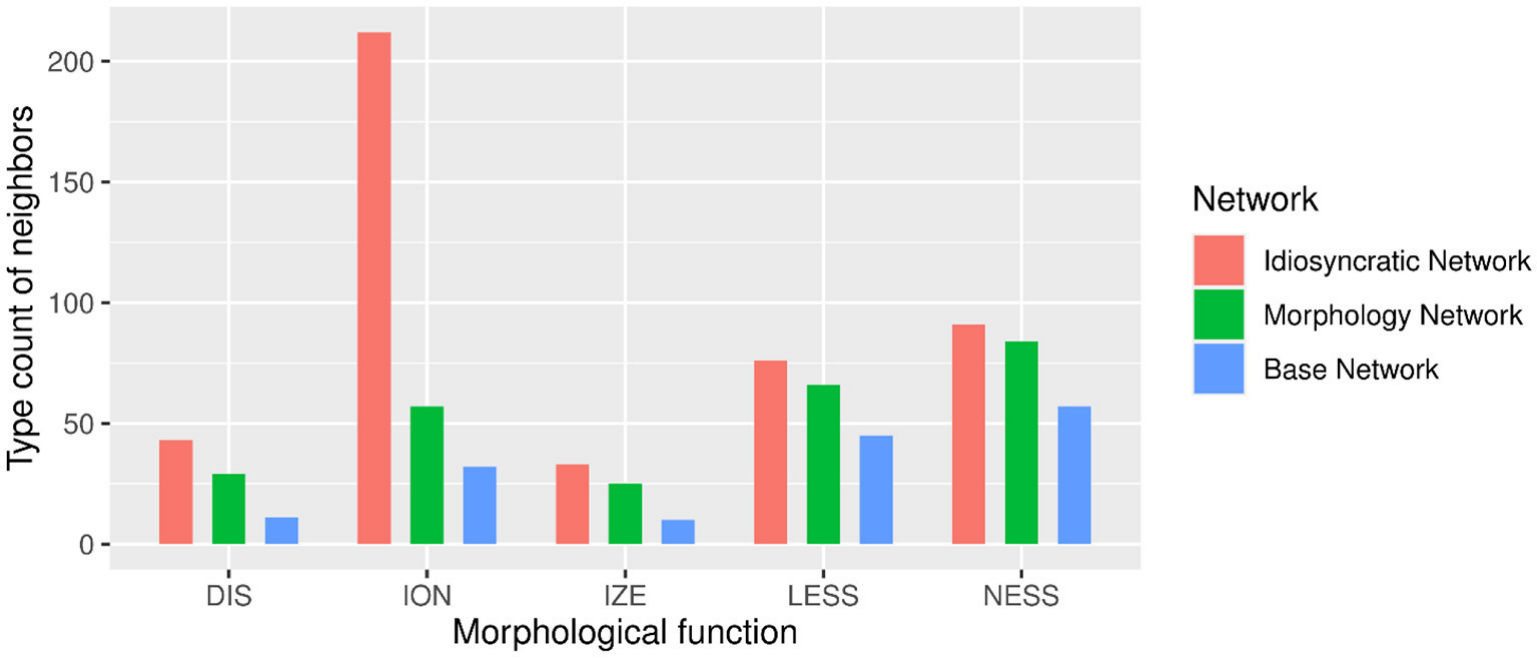
Type count of top 8 neighbors by network and morphological function.

Exploring the aberrant behavior of the Base Network further, we investigated the semantic space of the Base Network in more detail and found that the clustering of words in the semantic space is detrimental. This is exemplified in Table 7, which shows an extract from the list of closest semantic neighbors to words with dis in the three networks. Quite expectedly, the Idiosyncratic Network features a lower number of dis words as neighbors of target dis words than the other two networks. And there are more neighbors featuring dis in the Base Network than in the Morphology Network. This increase of the number of dis words as neighbors across the three networks mirrors the increasing role of explicit morphological information encoded in these networks. There is an important difference, however, between the Morphology Network and the Base Network. While in the Morphology Network, the dis neighbors consist of many different word types with dis, in the Base Network these are very often exactly the same word types. A type analysis of the neighbors for all morphological categories in the three networks confirms this impression: Figure 6 shows that the Base Network is characterized by the least diverse neighbor space of the three networks, and that this is true for every investigated morphological function. Given this behavior, it is thus no longer surprising that measures derived from the Base Network might behave strangely or not display effects. We conclude that the Base Network is not only theoretically the least appealing of the three networks, but that these problems also lead to an empirically unattractive model.

**Table 7:** Extract from the closest semantic neighbors of DIS words in the three networks.

**Word**	**Phones**	**Neighbors**						
**Idiosyncratic network**								
disarm	dIs,m	m1d1	kInt	w{m	m{mb5	kr{NkI	n5zI	bl{NklI
disband	dIsb{nd	m1d1	kInt	bl{NklI	w{m	m{mb5	kr{NkI	pIpIn
discard	dIsk,d	dIs@r1	dIst1st	dIskrEdIt	dIsgr1s	dIskVmf@t	$l	dIs@b1
discharge	dIsJ,=	dIsl2k	dIsQnIst	dIstrVst	dIs@gri	dIskVmf@t	dIsgr1s	dIsk@ntEnt
disclose	dIskl5z	m1d1	kInt	m{mb5	w{m	bl{NklI	n5zI	SIt
discount	dIsk6nt	dIsQnIst	dIskVmf@t	dIsgr1s	dIsk@ntEnt	dIstrVst	dIst1st	dIsg2z
discourse	dIsk$s	dIs@r1	dIst1st	dIskrEdIt	dIsgr1s	dIskVmf@t	dIsp{r@tI	dIslQ=
disease	dIziz	dIskVv@R	dIs@p7R	dIs$d@R	dIsJ,=	dIsl2k	dIsk6nt	dIs@gri
disgrace	dIsgr1s	dIst1st	dIskVmf@t	dIs@r1	dIskrEdIt	dIs@b1	dIslQ=	dIsp{r@tI
disguise	dIsg2z	dIskVmf@t	dIsgr1s	dIst1st	dIs@r1	dIsQnIst	dIsk@ntEnt	dIskrEdIt
dislike	dIsl2k	dIsQnIst	dIskVmf@t	dIsgr1s	dIstrVst	dIsk@ntEnt	dIst1st	dIsg2z
**Morphology network**								
disarm	dIs,m	dIsjun@tI	dIs5n	dIsb{nd	dIs@r1	dIskrEdIt	dIsp{r@tI	dIs@b1
disband	dIsb{nd	dIsjun@tI	dIs5n	dIs,m	dIs@r1	dIskrEdIt	dIs@b1	dIsp{r@tI
discard	dIsk,d	dIskVmf@t	dIsgr1s	dIst1st	dIsQnIst	dIs@r1	dIsk@ntEnt	dIslQ=
discharge	dIsJ,=	dIsl2k	dIsQnIst	dIstrVst	dIs@gri	dIskVmf@t	dIsgr1s	dIsk@ntEnt
disclose	dIskl5z	dIs@r1	dIs5n	dIs,m	dIskrEdIt	dIsjun@tI	dIsb{nd	dIsp{r@tI
discount	dIsk6nt	dIskVmf@t	dIsQnIst	dIsgr1s	dIsl2k	dIs@gri	dIstrVst	dIsg2z
discourse	dIsk$s	dIskVmf@t	dIsgr1s	dIst1st	dIsQnIst	dIsk@ntEnt	dIs@r1	dIsrIg,d
disease	dIziz	dIskVv@R	dIs@p7R	dIs$d@R	dIsJ,=	dIsl2k	dIsk6nt	dIs@gri
disgrace	dIsgr1s	dIst1st	dIskVmf@t	dIs@r1	dIskrEdIt	dIs@b1	dIslQ=	dIsp{r@tI
disguise	dIsg2z	dIskVmf@t	dIsgr1s	dIst1st	dIs@r1	dIsQnIst	dIsk@ntEnt	dIskrEdIt
dislike	dIsl2k	dIskVmf@t	dIsQnIst	dIsgr1s	dIstrVst	dIs@gri	dIsk@ntEnt	dIsg2z
**Base network**								
disarm	dIs,m	dIsg2z	dIsp{r@tI	dIsgVst	dIs@r1	dIsl2k	dIs@bidj@ns	dIspl1s
disband	dIsb{nd	dIsg2z	dIsp{r@tI	dIs@r1	dIsgVst	dIsl2k	dIspl1s	dIs@bidj@ns
discard	dIsk,d	dIsg2z	dIsp{r@tI	dIsgVst	dIs@r1	dIs@bidj@ns	dIsl2k	dIspl1s
discharge	dIsJ,=	dIsg2z	dIsp{r@tI	dIsgVst	dIs@r1	dIsl2k	dIs@bidj@ns	dIsQnIst
disclose	dIskl5z	dIsg2z	dIsp{r@tI	dIsgVst	dIs@r1	dIsl2k	dIs@bidj@ns	dIslQ=
discount	dIsk6nt	dIsg2z	dIsp{r@tI	dIsgVst	dIs@r1	dIs@bidj@ns	dIsl2k	dIsQnIst
discourse	dIsk$s	dIsg2z	dIsp{r@tI	dIsgVst	dIs@r1	dIspl1s	dIs@bidj@ns	dIsQnIst
disease	dIziz	dIsg2z	dIsp{r@tI	dIsgVst	dIs@r1	dIs@bidj@ns	dIsl2k	dIsQnIst
disgrace	dIsgr1s	dIsg2z	dIsp{r@tI	dIsgVst	dIs@r1	dIsl2k	dIs@bidj@ns	dIsQnIst
disguise	dIsg2z	dIsp{r@tI	dIsgVst	dIs@r1	dIs@bidj@ns	dIsl2k	dIsQnIst	dIslQ=
dislike	dIsl2k	dIsg2z	dIsp{r@tI	dIsgVst	dIs@r1	dIs@bidj@ns	dIslQ=	dIsQnIst

## Discussion and Conclusion

This study set out to explore how morphological effects on the phonetic output, which have been frequently observed in the literature, can be explained. From the perspective of current speech production models and theories of the morphology-phonology interaction, such effects are unexpected, and the mechanisms behind them are unclear. Our study investigated whether we can successfully model the durations of English derivatives with a new psycho-computational approach, linear discriminative learning. We hypothesized that measures derived from an LDL network are predictive of duration. We also explored what insight their effects can give us into the mechanisms of speech production, and whether the measures derived from these networks differ in their predictive power depending on the kind of information they have about morphological functions.

Our study demonstrated that LDL-derived variables can successfully predict derivative durations. The mean semantic support of a word's articulatory path, the entropy of a word's path supports, the mean correlation of a word's predicted semantics with the semantics of its neighbors, and the distance of the semantic vector in the semantic space all significantly affect duration. We have also shown that these measures explain a reasonable proportion of the durational variance, in the sense that their contribution to the explained variance is comparable to the contribution of traditional linguistic variables used in corpus studies of duration. The present study thus contributes to the growing literature that demonstrates that LDL is a promising alternative approach to speech production which can explain the variation in fine phonetic detail we find in different kinds of words, be they simplex, complex, or non-words (cf. Baayen et al., 2019b; Chuang et al., 2020).

Regarding the question what the effects of LDL-derived variables can tell us about speech production, we find that two important concepts relevant for production are the certainty in the association of form with meaning and the semantic relations of words to other words. The positive effects of mean word support and the negative effects of path entropies on duration both indicate that generally, higher certainty in the association of form and meaning is associated with longer durations. The better an articulatory path is on average semantically supported, and the less these supports vary over the path, the more strengthened the articulation becomes. It is important to note that the metaphor of “certainty” which is ascribed to these measures can generate two opposing expectations, both of which are intuitive in their own way. On the one hand, it could be assumed that the more certain a speaker is, the more strengthened the signal will be, leading to longer durations. This may be because a speaker invests more energy in maintaining duration when they are certain, and less energy when they are uncertain, in order to not prolong a state of uncertainty (Tucker et al., 2019). On the other hand, it could be assumed that the more certain a speaker is, the more efficient they can articulate, leading to shorter durations. This may be because more certainty could enable a speaker to select the correct path more quickly. The present results provide support for the first interpretation rather than the second one.

This interpretation is in line with the findings for other measures that have been interpreted with reference to the concept of certainty. Tomaschek et al. (2019), for instance, found that with higher functional certainty, gauged by the support for a word's inflectional lexome and the word's overall baseline support, segment durations of different types of English final S are lengthened. Kuperman et al. (2007) found that with higher certainty, gauged by the paradigmatic support (probability) of Dutch compound interfixes, these interfixes are realized longer. Cohen (2014) found that higher certainty, gauged by the paradigmatic probability of English suffixes, is associated with phonetic enhancement, i.e., again with longer durations. Cohen (2015) found that higher paradigmatic support can also enhance Russian vowels. Tucker et al. (2019) found that with higher support for tense and regularity (more certainty), acoustic duration of stem vowels increases, and with greater activation diversity (more uncertainty), acoustic duration decreases. In sum, regarding the question whether certainty has an effect of enhancement or reduction, recent evidence—including the present study—points toward enhancement.

The significant effects of semantic density and of semantic vector length indicate that a second relevant factor in the production of derivatives is the semantic relation of a word to other words. Starting with semantic density, depending on the architecture of the network, the average semantic similarity of a word's neighbors to this word can lead to both longer and shorter durations. If the network has information about the semantics of the morphological category of the derivative and of the derivative itself, higher densities are associated with longer durations. If the network has no such information and treats all words as idiosyncratic, or if the network has information about the morphological category and the semantics of the derivative's base word, higher densities are associated with shorter durations. In order to get a better understanding of this somewhat puzzling finding, three observations are helpful.

Let us first compare the Idiosyncratic Network and the Morphology Network. We can see in the data points in Figure 3 as well as in the density plots in Figure 5 that semantic density is distributed very differently when derived from the Idiosyncratic Network than when derived from the Morphology Network (both the model results as well as the distributions are plotted on the same x-axis scale, respectively, for easier comparison). For the Idiosyncratic Network, there are hardly any data points above 0.8 and the vast majority of data points have density values below 0.4. For the Morphology Network, on the other hand, the vast majority of data points show densities above 0.8. At the conceptual level this makes sense: We would expect words sharing the semantics of their morphological category to be closer to their neighboring words, i.e., to be more transparent and less idiosyncratic. This means that if the model has information about morphological categories, density should be generally higher. This is the case. In contrast, words in the Idiosyncratic Network are generally more dissimilar to each other because they do not share the semantic information that comes with belonging to a particular morphological category. This difference between the two networks is also illustrated by the example of dis neighbors in Table 7, which shows that in the Morphology Network a larger proportion of nearest neighbors comes from the morphological category of the target word.

Returning to the relation between semantic density and duration, we can now see in Figure 3 that the contradictory effects happen at different ends of the distribution. The negative effect found in the Idiosyncratic Network is carried by the low-density words, while the positive effect of semantic density on duration is carried by the high-density words. The positive effect of densities above 0.8 is even visible in the Idiosyncratic Network: the residuals in that range are clearly skewed toward higher durations. If we attempt an interpretation of the relation of semantic density and word duration across these two networks, we can say that the shortest durations are found in the middle of the semantic density range. Having many close semantic relatives slows down articulation, and so does having very few relatives.

What about semantic density in the Base Network? semantic density in this network is distributed similarly to the Morphology Network, yet the effect is similar to the Idiosyncratic Network, as it negatively affects duration. However, our exploration of the type diversity in the semantic space of the networks in the section Semantic Density and Semantic Vector Length has shown that the neighbors that are behind these semantic densities are not at all diverse in the Base Network. This was true to such an extent that for the dis words, for example, the Base Network considered the same few words (especially *disguise, disparity, disgust, disarray*) to be the closest neighbors for the vast majority of the target words. We consider this clustering to be rather unrealistic. Most likely, it is the consequence of the questionable premises underlying this network architecture discussed earlier. Overall, we conclude that the effect of semantic density in this network is not interpretable.

The question remains how we can understand the opposite effects of semantic density in the Idiosyncratic Network and the Morphology Network. If our interpretation that semantic density captures semantic transparency is correct, we would expect higher densities to lead to longer durations. More transparent words should be more protected against phonetic reduction because they feature a stronger morphological “boundary,” i.e., they are more decomposable. Such lengthening effects induced by supposed morphological boundaries have been observed in several studies (e.g., Hay, 2001, 2003, 2007; Plag and Ben Hedia, 2018). If we assume that the theoretical concept of a morphological boundary and the similarity of a word to its neighboring words capture the same underlying dimension of semantic transparency, we should still be able to replicate this effect. However, it is not entirely clear why a higher degree of semantic transparency would lead to lengthening. Given that a higher semantic transparency means that more words will be more strongly activated, we would rather expect durations to shorten. This is because semantic activation diversity has been found to be associated with reduction (Tucker et al., 2019). This reduction in speech production is mirrored in reaction time experiments that have found shorter reaction times with larger morphological family sizes (Schreuder and Baayen, 1997; Bertram et al., 2000). This family size effect has been interpreted as a semantic effect arising through activation spreading between morphologically related words. Interestingly, in the study by Bertram et al. (2000), the effect was restricted to transparent family members. This is an indication that the effect may not be as linear as standardly assumed.

A non-linear, *U*-shaped effect of transparency on reaction times was observed by Plag and Baayen (2009). These authors demonstrated that suffixes that are either very easily segmentable or hardly segmentable have lower processing costs (as gauged by shorter reaction times in lexical decision) than suffixes in the middle of the segmentability range. Plag and Baayen (2009) interpreted this as an effect of the opposing forces of storage and computation. Assuming that our high-density words are those that are easily segmentable, while our low-density words are the ones that are not segmentable, we can come up with the tentative interpretation that the short durations in the mid-range of density are a reflection of the higher processing costs incurred by the forms in the middle of the segmentability scale. One problem with this account is, however, that higher processing costs in lexical decision seem to be correlated with shorter durations in production, but with longer latencies in comprehension. This contradiction can only be solved if we know more about the specific processing differences between production and comprehension, or about the specific processing stages involved in lexical decision vs. freely generated conversational speech. We leave this issue to be explored in future studies.

The second variable capturing the semantic relation between words that this study has shown to be able to successfully predict duration is semantic vector length. Compared to semantic density, the effect of semantic vector length is more straightforward to interpret. A longer semantic vector, i.e., a higher activation diversity, is associated with shorter duration. Tucker et al. (2019) argue that the more semantic dimensions a speaker is active on for a word, the more confusable the meanings of this word are. When more meanings are activated and these meanings are more confusable, the speaker is more uncertain and therefore invests less energy in maintaining the signal. In this account, our finding that words with higher activation diversity are shorter is thus expected.

Let us now return to the role of morphological information in our networks. Importantly, our results for the two semantic variables show that differences between morphological categories can emerge even from the network without any information about derivational functions. For example, semantic density is significantly higher for words with the derivational functions ness, less and dis than for words with ation. This is in accordance with traditional descriptions of the semantic transparency of affixes, which posit *-ness, -less*, and *dis-* as producing mostly transparent derivatives, while words with*-ation* are assumed to be less transparent (Bauer et al., 2013; Plag, 2018). Only ize does not fit that pattern, as many ize words are characterized by high densities but are considered about as transparent as *-ation* (however, *-ize* is considered to be more productive than *-ation*). Another interesting example of this is the distribution of semantic vector length. The longer the vector of a word, the higher its semantic activation diversity becomes and the more collocational relations it has to other words, i.e., the more polysemous it is. The average vector length was highest for ize and ation words. This reflects traditional descriptions of *-ize* and *-ation* having highly multifaceted semantics (cf. the locative, ornative, causative, resultative, inchoative, performative or similative meaning of *-ize*, and the meanings of *-ation* denoting events, states, locations, products, or means; Bauer et al., 2013; Plag, 2018). The affixes *-less, dis-*, and to a lesser extent *-ness*, on the other hand, have comparatively clearer and narrower semantics. In sum, these differences between morphological categories in the Idiosyncratic Network demonstrate that LDL can discriminate derivational functions from sublexical and contextual cues alone.

Our results have implications for morphological theory and speech production models. First, the acoustic properties of morphologically complex words can be modeled successfully by implementing a discriminative learning approach. Traditional approaches were largely unable to accommodate effects of morphological structure on the phonetic output production (Chomsky and Halle, 1968; Kiparsky, 1982; Dell, 1986; Levelt et al., 1999; Roelofs and Ferreira, 2019; Turk and Shattuck-Hufnagel, 2020). Many theories of morphology-phonology interaction assume that morphological boundaries are erased in the process of passing morphemic units on to phonological processing. And many models of speech production assume an articulator module that realizes phonemic representations with pre-programmed gesture templates independently of morphemic status. These approaches lack explanations for the fact that a word's morphological structure or semantics can cause differences in articulatory gestures, as they do not allow for a direct morphology-phonetics interaction. In LDL, however, such interaction is expected and can be explained by its underlying theoretical principles of learning and experience.

Second, our implementations show that morphological functions can emerge as a by-product of a morpheme-free learning process. Morphology is possible without morphemes. Given the many problems with the morpheme as a theoretical construct (see, e.g., Baayen et al., 2019b), this is a welcome finding. Finding morphological effects on phonetic realization need not lead to the conclusion that these effects must originate from morphemes. They can also emerge in the mapping of forms and meanings that have no information on morphology at all (see, e.g., Baayen et al., 2011 et seq. for more examples of this). As Divjak (2019) puts it, “it is not because a phenomenon can be described in a certain way that the description is psychologically realistic, let alone real” (p. 247). Of course, the success of LDL in this study and others does not allow us to infer that there is no cognitive plausibility to these structural units at all. If LDL is modeling rather how children learn languages, adult speakers may learn differently once they have explicit knowledge of morphemic structure. Such structure might also be acquired after-the-fact, when a speaker has seen enough words to start seeing analogies, or after learning about this structure explicitly. The morpheme might be epiphenomenal rather than superfluous. However, LDL does demonstrate that such fixed units of form and meaning are at the very least not obligatory. The connection between form and meaning can be dynamic and relational, allowing morphological theory to reframe its semiotic legacy. In fact, it has been argued that since its discriminative underpinnings emphasize that language is a system of *différence*, discriminative learning elegantly carries the discipline back to its Saussurean heritage (Blevins, 2016).

There are several potential future directions for discriminative learning studies on the phonetics of derived words. First, it would be interesting to model the durations of more derivational functions in a larger dataset. Investigating more than the five morphological categories of the present study might reveal further important differences between these categories. Second, one issue that we would like to resolve in future studies concerns the response variable. In a corpus study of duration with different word types, it is essential to control for phonological length. This is why instead of duration, we decided to model duration difference, i.e., the residuals of a model regressing a word's absolute duration against the sum of its average segment durations. However, for an LDL implementation, this response variable is not optimal, since strictly speaking it still implicitly assumes segmental structure. It would be desirable to control for segmental makeup without actually having to refer to segments. Third, we think it could be fruitful to investigate how best to construct vectors for words with multiple derivational functions. This would enable us to gain more insight into the complex interplay of morphological categories. And, finally, we think that to further test how well LDL can predict durations when the semantics of derivatives are strictly compositional (like in the Base Network), one interesting avenue for future research would be to use vectors that already assume this compositionality when generating lexome-to-lexome vectors.^6^ That is, while in the present study we used lexome vectors that Baayen et al. (2019b) generated by using the Widrow-Hoff algorithm to predict function lexomes in addition to content lexomes for words in a sentence, it is conceivable to use vectors generated by predicting function lexomes in addition to content lexomes for *bases* in a sentence. The lexome vector $\vec{happy}$, for instance, would then capture relations to contextual lexomes surrounding the word *happiness* as well. Similarly, one could generate vectors of derived words to use in the Idiosyncratic Network that do not capture cues of any functional lexomes by refraining from coding them altogether in the training data. We leave this interesting option to be explored in future studies.

To summarize, this study modeled the acoustic duration of 4,530 English derivative tokens with the morphological functions dis, ness, less, ation, and ize in natural speech data, using predictors derived from a linear discriminative learning network. We have demonstrated that these measures can successfully predict derivative durations. They reveal that more semantic certainty in pronunciation is associated with acoustic enhancement, i.e., longer durations, which is consistent with previous studies of paradigmatic probability and semantic support measures. We have also shown that differences between morphological categories emerge from the network, even without explicitly providing the network with such information. This further strengthens the position of LDL as a promising theoretical alternative for speech production, and provides further evidence that morphology is possible without morphemes.

## Data Availability Statement

The data, scripts and materials used in this study can be found in an online repository at https://osf.io/jkncb.

## Author Contributions

IP and SS contributed to conception and design of the study. SS retrieved the data, performed the modeling and statistical analysis, and wrote the first draft of the manuscript. All authors contributed to manuscript revision, read, and approved the submitted version.

## Conflict of Interest

The authors declare that the research was conducted in the absence of any commercial or financial relationships that could be construed as a potential conflict of interest. The handling editor declared a past co-editorship with one of the authors IP.

## Publisher's Note

All claims expressed in this article are solely those of the authors and do not necessarily represent those of their affiliated organizations, or those of the publisher, the editors and the reviewers. Any product that may be evaluated in this article, or claim that may be made by its manufacturer, is not guaranteed or endorsed by the publisher.

## Footnotes

^1^The main diagonal of a lexome-to-lexome matrix represents the association strengths of each word to itself. Each word occurring in a sentence naturally predicts itself very well to occur in that sentence, but this value is not very informative about the word's relation to other words. Baayen et al. (2019b) therefore argue that when the researcher is interested in semantic similarity, they should replace these values with zero values.

^2^Note that the form matrices *C*_*I*_, *C*_*M*_, and *C*_*B*_ are identical, as the networks only differ in their construction of semantic vectors, not of form vectors.

^3^Correlation matrices and variable clustering trees for both LDL-derived variables and traditional variables are documented in the Supplementary Material.

^4^We used a different corpus than the Audio BNC for this task because the Audio BNC does not provide this information in an accessible and reliable form.

^5^We provide plots illustrating the frequency distribution in our data in the Supplementary Material.

^6^Thanks are due to Reviewer 2 for this suggestion.
