# Supplementary material for: Morpho-Phonetic Effects in Speech Production: Modeling the Acoustic Duration of English Derived Words With Linear Discriminative Learning
Source: Front Psychol. 2021 Aug 2;12:678712. doi: 10.3389/fpsyg.2021.678712 (PMC8366231; doi:10.3389/fpsyg.2021.678712)
Supplement: Supplementary file 1 [file Data_Sheet_1.pdf]

# Supplementary Tables

Supplementary Table 1: Descriptive statistics for all continuous variables. The letters M and I refer to the M-Network and the I-Network, respectively.

| | *Min.* | *1st Qu.* | *Median* | *3rd Qu.* | *Max.* | *SD* |
|---|---|---|---|---|---|---|
| DURATION DIFFERENCE | -0.438032 | -0.085213 | -0.009462 | 0.074622 | 0.676428 | 0.1256573 |
| MEAN WORD SUPPORT M | 0.7000 | 1.0000 | 1.0000 | 1.0000 | 1.0000 | 0.07070581 |
| MEAN WORD SUPPORT I | 0.3333 | 1.0000 | 1.0000 | 1.0000 | 1.0000 | 0.07135186 |
| PATH ENTROPIES M | 0.0000 | 0.0000 | 0.9183 | 1.3690 | 2.5265 | 0.641389 |
| PATH ENTROPIES I | 0.0000 | 0.0000 | 0.9183 | 1.3690 | 2.5265 | 0.641389 |
| SEMANTIC VECTOR LENGTH M | 1.770 | 2.290 | 2.460 | 2.710 | 3.090 | 0.256338 |
| SEMANTIC VECTOR LENGTH I | 0.06171 | 0.74697 | 1.38993 | 2.03160 | 2.43150 | 0.659131 |
| SEMANTIC DENSITY M | 0.5720 | 0.7783 | 0.8808 | 0.9655 | 0.9990 | 0.1227957 |
| SEMANTIC DENSITY I | 0.1825 | 0.2723 | 0.3600 | 0.4472 | 0.9048 | 0.1842267 |
| TARGET CORRELATION M | 0.7905 | 0.9866 | 1.0000 | 1.0000 | 1.0000 | 0.0166425 |
| TARGET CORRELATION I | 0.2242 | 0.9864 | 1.0000 | 1.0000 | 1.0000 | 0.1056093 |
| SPEECH RATE | 2.632 | 5.325 | 6.098 | 6.897 | 11.348 | 1.21582 |
| WORD FREQUENCY | 81 | 4473 | 10423 | 29362 | 137034 | 40697.69 |
| RELATIVE FREQUENCY | 0.0025 | 0.0681 | 0.6277 | 3.9972 | 819.4094 | 36.03326 |
| MEAN BIPHONE PROBABILITY | 0.0005667 | 0.0028750 | 0.0040889 | 0.0053778 | 0.0102857 | 0.001741725 |
| BIGRAM FREQUENCY | 1 | 1 | 45 | 587 | 12216 | 2329.012 |

Supplementary Table 2: Count of levels for the categorical variable AFFIX.

| AFFIX | *less* | *ness* | *ation* | *dis* | *ize* |
|---|---|---|---|---|---|
| observations | 145 | 344 | 3403 | 233 | 405 |

Supplementary Table 3: Final standard linear regression model reporting effects on duration difference with variables from the Idiosyncratic Network. Significance codes: *** < 0.001, ** < 0.01, * < 0.05.

| | *Estimate* | *Std. Err.* | *t-value* | *Pr(>\|t\|)* | |
|---|---|---|---|---|---|
| Intercept | 0.216901 | 0.026210 | 8.276 | < 2e-16 | *** |
| MEAN WORD SUPPORT | 0.170726 | 0.023507 | 7.263 | 4.45e-13 | *** |
| PATH ENTROPIES | -0.008688 | 0.002242 | -3.875 | 0.000108 | *** |
| SEMANTIC DENSITY | -0.043545 | 0.008925 | -4.879 | 1.10e-06 | *** |
| SPEECH RATE | -0.058757 | 0.001148 | -51.186 | < 2e-16 | *** |
| | *$R^2$ multiple: 0.3784, adjusted: 0.3778* | | | | |

Supplementary Table 4: Final standard linear regression model reporting effects on duration difference with variables from the Morphology Network. Significance codes: *** < 0.001, ** < 0.01, * < 0.05.

| | *Estimate* | *Std. Err.* | *t-value* | *Pr(>\|t\|)* | |
|---|---|---|---|---|---|
| Intercept | 0.090708 | 0.025887 | 3.504 | 0.000463 | *** |
| MEAN WORD SUPPORT | 0.250262 | 0.020700 | 12.090 | < 2e-16 | *** |
| PATH ENTROPIES | -0.008442 | 0.002309 | -3.656 | 0.000259 | *** |
| SEMANTIC DENSITY | 0.033868 | 0.012372 | 2.737 | 0.006217 | ** |
| SPEECH RATE | -0.058602 | 0.001159 | -50.579 | < 2e-16 | *** |
| | *$R^2$ multiple: 0.3748, adjusted: 0.3742* | | | | |

Supplementary Table 5: Final standard linear regression model reporting effects on duration difference with variables from the Base Network. Significance codes: *** < 0.001, ** < 0.01, * < 0.05.

| | *Estimate* | *Std. Err.* | *t-value* | *Pr(>\|t\|)* | |
|---|---|---|---|---|---|
| Intercept | 0.408246 | 0.029999 | 13.609 | < 2e-16 | *** |
| MEAN WORD SUPPORT | 0.050723 | 0.012716 | 3.989 | 6.75e-05 | *** |
| PATH ENTROPIES | -0.009342 | 0.002259 | -4.135 | 3.61e-05 | *** |
| SEMANTIC DENSITY | -0.093906 | 0.025844 | -3.634 | 0.000283 | *** |
| SPEECH RATE | -0.058702 | 0.001171 | -50.138 | < 2e-16 | *** |
| | *$R^2$ multiple: 0.3629, adjusted: 0.3623* | | | | |

Supplementary Table 6: Final mixed-effects regression model reporting effects on duration difference with variables from the Idiosyncratic Network. Significance codes: *** < 0.001, ** < 0.01, * < 0.05.

| | *Estimate* | *Std. Err.* | *df* | *t-value* | *Pr(>\|t\|)* | |
|---|---|---|---|---|---|---|
| Intercept | 1.328e-01 | 4.601e-02 | 2.567e+02 | 2.887 | 0.00422 | ** |
| MEAN WORD SUPPORT | 2.722e-01 | 4.600e-02 | 2.535e+02 | 5.919 | 1.05e-08 | *** |
| PATH ENTROPIES | -1.173e-02 | 5.625e-03 | 2.387e+02 | -2.086 | 0.03807 | * |
| SEMANTIC VECTOR LENGTH | -1.606e-02 | 6.860e-03 | 1.968e+02 | -2.342 | 0.02018 | * |
| SPEECH RATE | -5.944e-02 | 1.116e-03 | 4.309e+03 | -53.281 | < 2e-16 | *** |
| | *$R^2$ marginal: 0.3690016, conditional: 0.5198377* | | | | | |

Supplementary Table 7: Final mixed-effects regression model reporting effects on duration difference with variables from the Morphology Network. Significance codes: *** < 0.001, ** < 0.01, * < 0.05.

| | *Estimate* | *Std. Err.* | *df* | *t-value* | *Pr(>\|t\|)* | |
|---|---|---|---|---|---|---|
| Intercept | 2.146e-01 | 6.024e-02 | 2.349e+02 | 3.563 | 0.000445 | *** |
| MEAN WORD SUPPORT | 2.535e-01 | 4.572e-02 | 2.538e+02 | 5.544 | 7.41e-08 | *** |
| PATH ENTROPIES | -1.163e-02 | 5.633e-03 | 2.380e+02 | -2.065 | 0.040022 | * |
| SEMANTIC VECTOR LENGTH | -3.294e-02 | 1.550e-02 | 2.035e+02 | -2.125 | 0.034752 | * |
| SPEECH RATE | -5.937e-02 | 1.116e-03 | 4.310e+03 | -53.202 | < 2e-16 | *** |
| | *$R^2$ marginal: 0.3638608, conditional: 0.5168201* | | | | | |

Supplementary Table 8: Final mixed-effects regression model reporting effects on duration difference with variables from the Base Network. Significance codes: *** < 0.001, ** < 0.01, * < 0.05.

| | *Estimate* | *Std. Err.* | *df* | *t-value* | *Pr(>\|t\|)* | |
|---|---|---|---|---|---|---|
| Intercept | 2.595e-01 | 2.510e-02 | 2.531e+02 | 10.341 | < 2e-16 | *** |
| MEAN WORD SUPPORT | 1.211e-01 | 2.654e-02 | 2.306e+02 | 4.562 | 8.24e-06 | *** |
| SPEECH RATE | -5.936e-02 | 1.117e-03 | 4.293e+03 | -53.121 | < 2e-16 | *** |
| | *$R^2$ marginal: 0.3487138, conditional: 0.5200542* | | | | | |

Supplementary Table 9: Standard linear regression model reporting effects on duration difference with traditional, non-LDL predictors. Significance codes: *** < 0.001, ** < 0.01, * < 0.05.

| | *Estimate* | *Std. Err.* | *t-value* | *Pr(>\|t\|)* | |
|---|---|---|---|---|---|
| Intercept | 3.888e-01 | 8.345e-03 | 46.589 | < 2e-16 | *** |
| WORD FREQUENCY | 4.970e-08 | 3.764e-08 | 1.320 | 0.187 | |
| RELATIVE FREQUENCY | -2.136e-05 | 4.166e-05 | -0.513 | 0.608 | |
| BIGRAM FREQUENCY | -6.542e-07 | 6.293e-07 | -1.039 | 0.299 | |
| MEAN BIPHONE PROBABILITY | -5.188e+00 | 8.872e-01 | -5.848 | 5.33e-09 | *** |
| AFFIX ation | | | | | |
| dis | 8.145e-03 | 6.700e-03 | 1.216 | 0.224 | |
| ize | -2.316e-02 | 5.251e-03 | -4.410 | 1.06e-05 | *** |
| less | -5.749e-02 | 8.226e-03 | -6.988 | 3.20e-12 | *** |
| ness | -5.473e-02 | 5.700e-03 | -9.601 | < 2e-16 | *** |
| SPEECH RATE | -5.893e-02 | 1.163e-03 | -50.670 | < 2e-16 | *** |
| | *$R^2$ multiple: 0.3744, adjusted: 0.3731* | | | | |

Supplementary Table 10: Mixed-effects regression model reporting effects on duration difference with traditional, non-LDL predictors. Significance codes: *** < 0.001, ** < 0.01, * < 0.05.

| | *Estimate* | *Std. Err.* | *df* | *t-value* | *Pr(>\|t\|)* | |
|---|---|---|---|---|---|---|
| Intercept | 4.159e-01 | 1.106e-02 | 1.433e+03 | 37.606 | < 2e-16 | *** |
| WORD FREQUENCY | -2.608e-07 | 2.328e-07 | 1.364e+02 | -1.120 | 0.26457 | |
| RELATIVE FREQUENCY | -1.446e-05 | 8.931e-05 | 3.397e+02 | -0.162 | 0.87149 | |
| BIGRAM FREQUENCY | 7.978e-07 | 6.382e-07 | 4.248e+03 | 1.250 | 0.21134 | |
| MEAN BIPHONE PROBABILITY | -7.167e+00 | 1.545e+00 | 1.589e+03 | -4.639 | 3.78e-06 | *** |
| AFFIX ation | | | | | | |
| dis | -1.405e-03 | 1.438e-02 | 2.667e+02 | -0.098 | 0.92227 | |
| ize | -1.491e-02 | 1.377e-02 | 2.542e+02 | -1.083 | 0.27981 | |
| less | -7.569e-02 | 1.524e-02 | 2.838e+02 | -4.968 | 1.17e-06 | *** |
| ness | -3.630e-02 | 1.295e-02 | 2.473e+02 | -2.803 | 0.00547 | ** |
| SPEECH RATE | -5.986e-02 | 1.116e-03 | 4.292e+03 | -53.633 | < 2e-16 | *** |
| | *R2 marginal: 0.3705799, conditional: 0.5344904* | | | | | |

Supplementary Table 11: Anova for standard regression model reporting effects on duration difference with traditional, non-LDL predictors. Significance codes: *** < 0.001, ** < 0.01, * < 0.05.

| | *Df* | *Sum Sq* | *Mean Sq* | *F-value* | *Pr(>F)* | |
|---|---|---|---|---|---|---|
| WORD FREQUENCY | 1 | 0.076 | 0.0761 | 8.8374 | 0.002967 | ** |
| RELATIVE FREQUENCY | 1 | 0.033 | 0.0329 | 3.8243 | 0.050577 | . |
| BIGRAM FREQUENCY | 1 | 0.062 | 0.0623 | 7.2354 | 0.007175 | ** |
| MEAN BIPHONE PROBABILITY | 1 | 0.022 | 0.0222 | 2.5741 | 0.108698 | |
| AFFIX | 4 | 0.577 | 0.1442 | 16.7515 | 1.23e-13 | *** |
| SPEECH RATE | 1 | 22.094 | 22.0938 | 2567.4015 | < 2.2e-16 | *** |

Supplementary Table 12: Anova for mixed-effects regression model reporting effects on duration difference with traditional, non-LDL predictors. Significance codes: *** < 0.001, ** < 0.01, * < 0.05.

| | *Sum Sq* | *Mean Sq* | *NumDF* | *DenDF* | *F-value* | *Pr(>F)* | |
|---|---|---|---|---|---|---|---|
| WORD FREQUENCY | 0.0088 | 0.0088 | 1 | 136.4 | 1.2550 | 0.2646 | |
| RELATIVE FREQUENCY | 0.0002 | 0.0002 | 1 | 339.7 | 0.0262 | 0.8715 | |
| BIGRAM FREQUENCY | 0.0109 | 0.0109 | 1 | 4247.8 | 1.5627 | 0.2113 | |
| MEAN BIPHONE PROBABILITY | 0.1508 | 0.1508 | 1 | 1588.5 | 21.5239 | 3.781e-06 | *** |
| AFFIX | 0.2068 | 0.0517 | 4 | 265.5 | 7.3805 | 1.192e-05 | *** |
| SPEECH RATE | 20.1514 | 20.1514 | 1 | 4291.6 | 2876.5118 | < 2.2e-16 | *** |

# Supplementary Figures

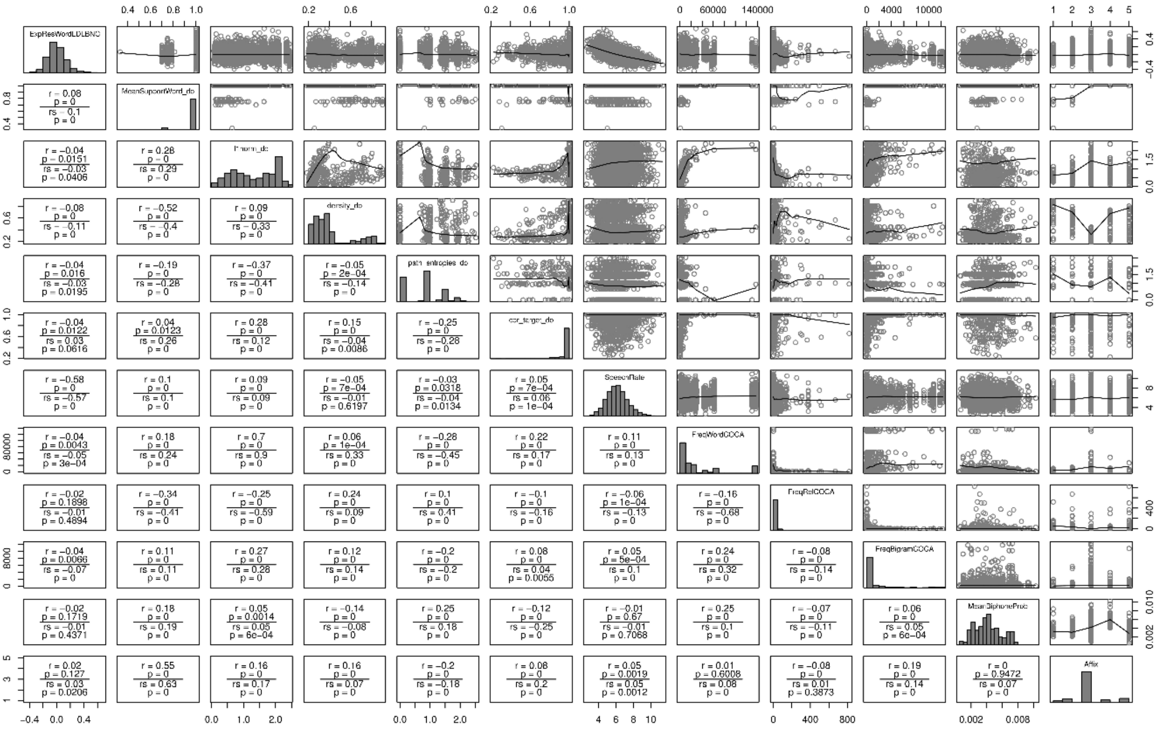


Supplementary Figure 1: Correlation matrix of traditional variables and LDL-derived variables in the Idiosyncratic Network.

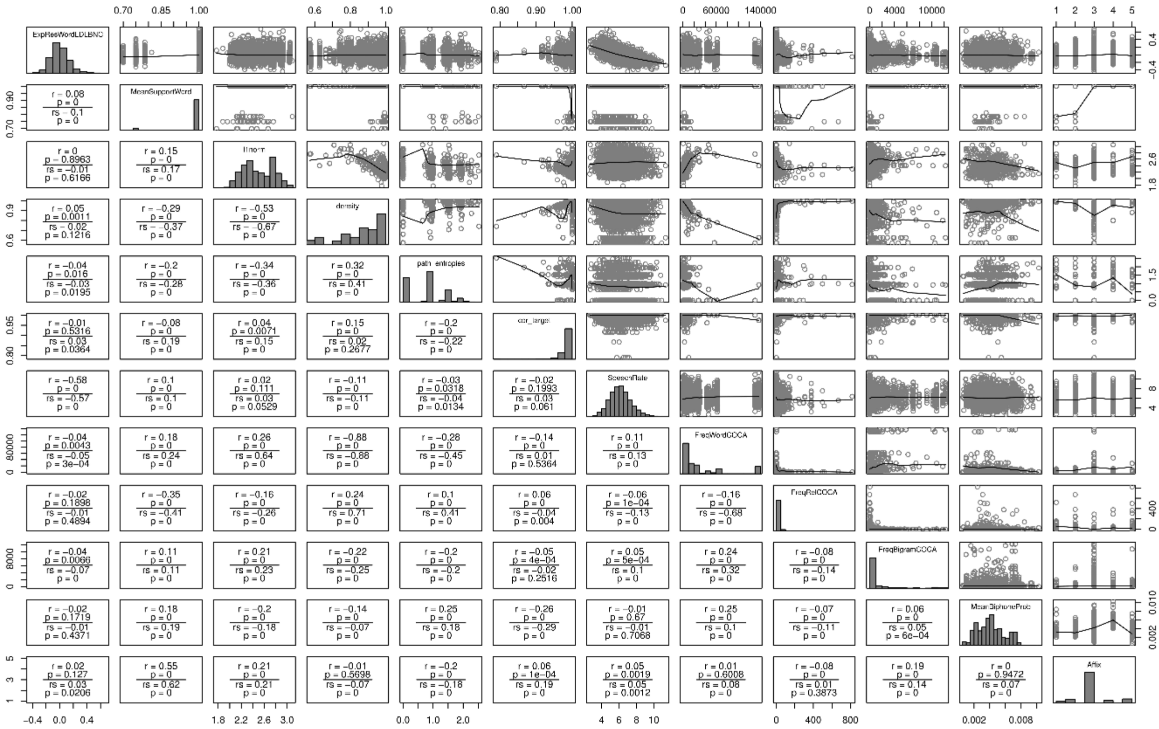


Supplementary Figure 2: Correlation matrix of traditional variables and LDL-derived variables in the Morphology Network.

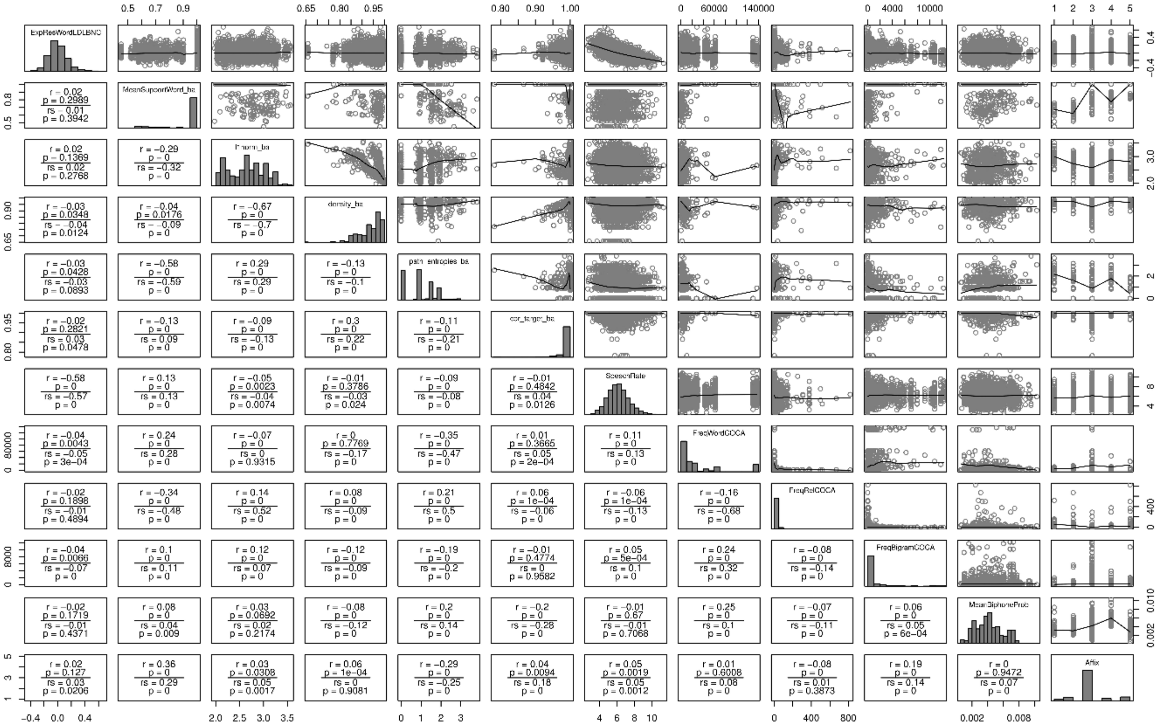

Supplementary Figure 3: Correlation matrix of traditional variables and LDL-derived variables in the Base Network.

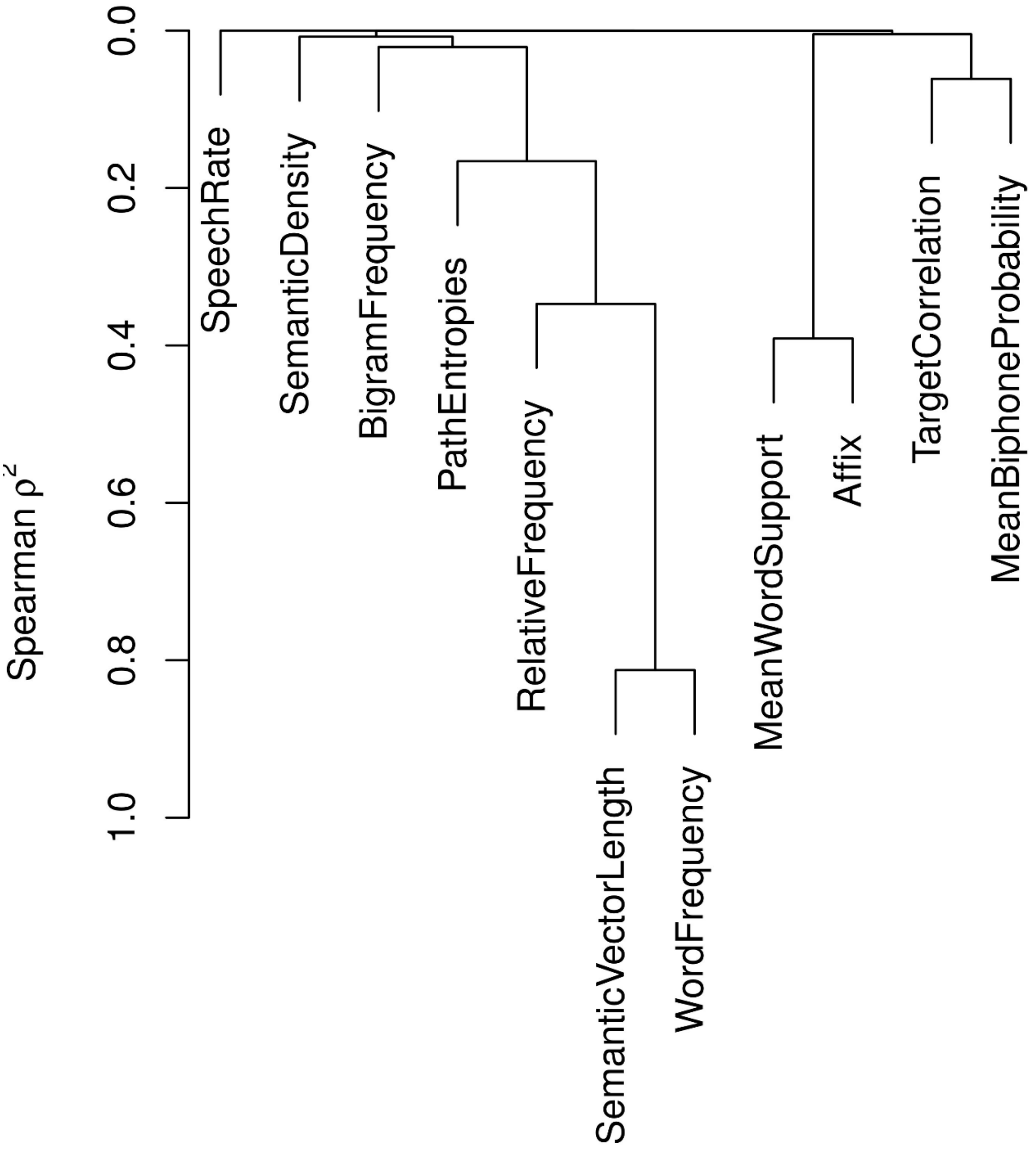


Supplementary Figure 4: Variable clustering tree of traditional variables and LDL-derived variables in the Idiosyncratic Network.

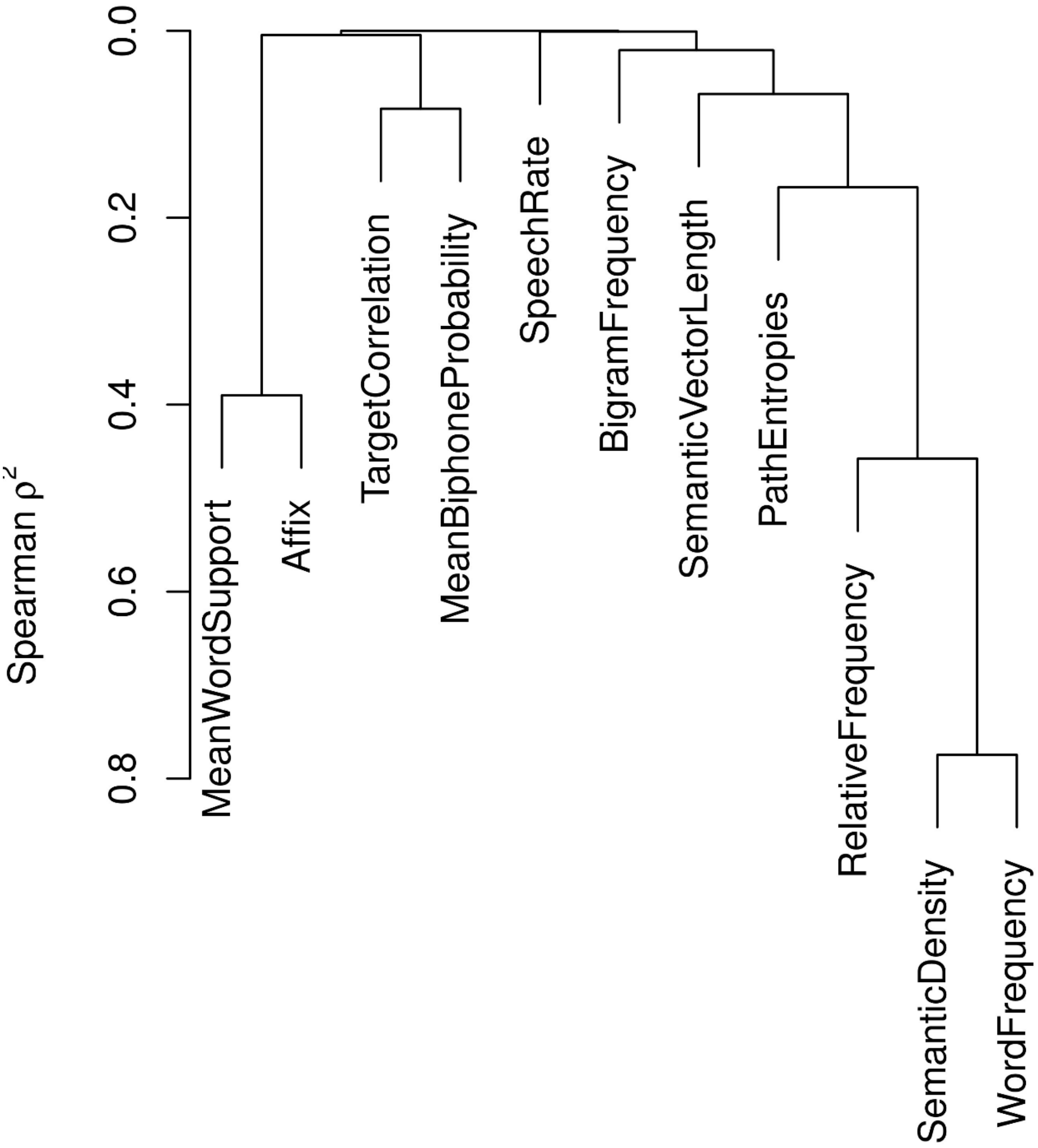


Supplementary Figure 5: Variable clustering tree of traditional variables and LDL-derived variables in the Morphology Network.

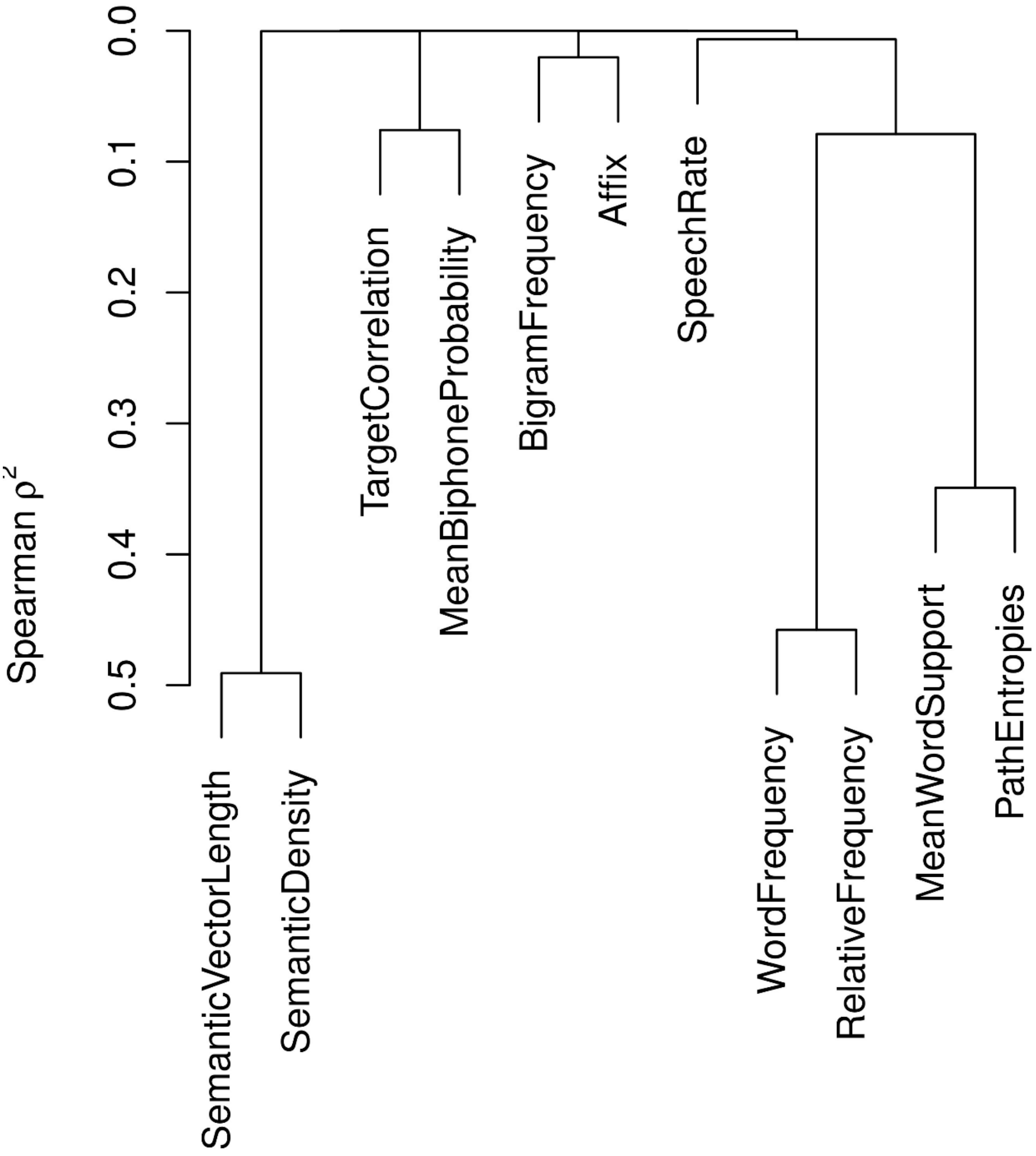


Supplementary Figure 6: Variable clustering tree of traditional variables and LDL-derived variables in the Base Network.

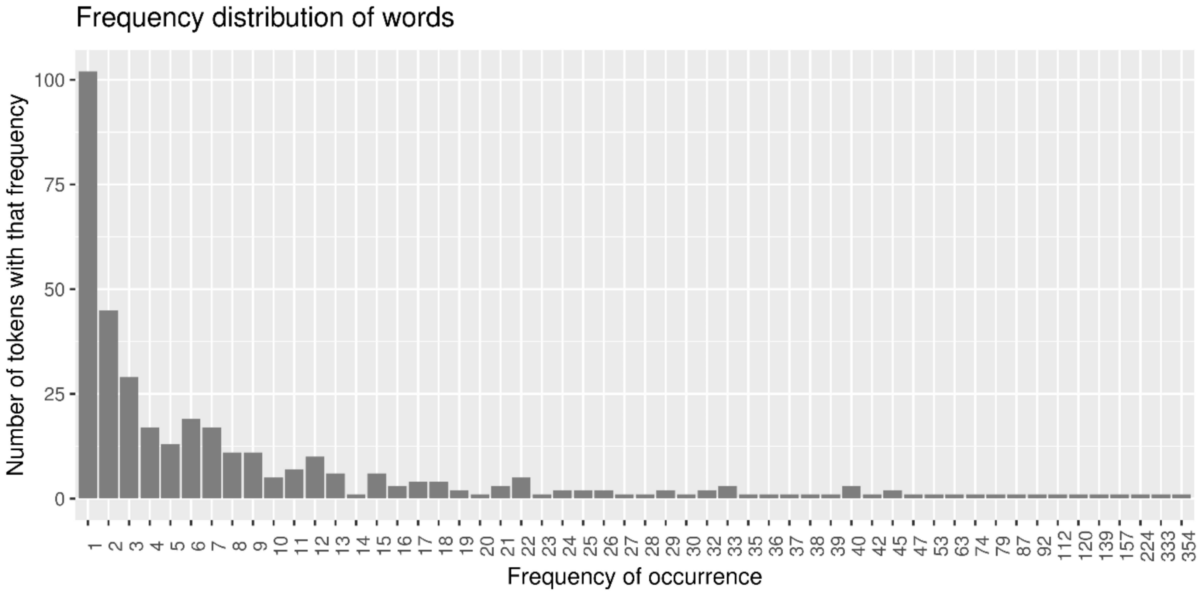


Supplementary Figure 7: Frequency of frequencies in the final Audio BNC dataset.

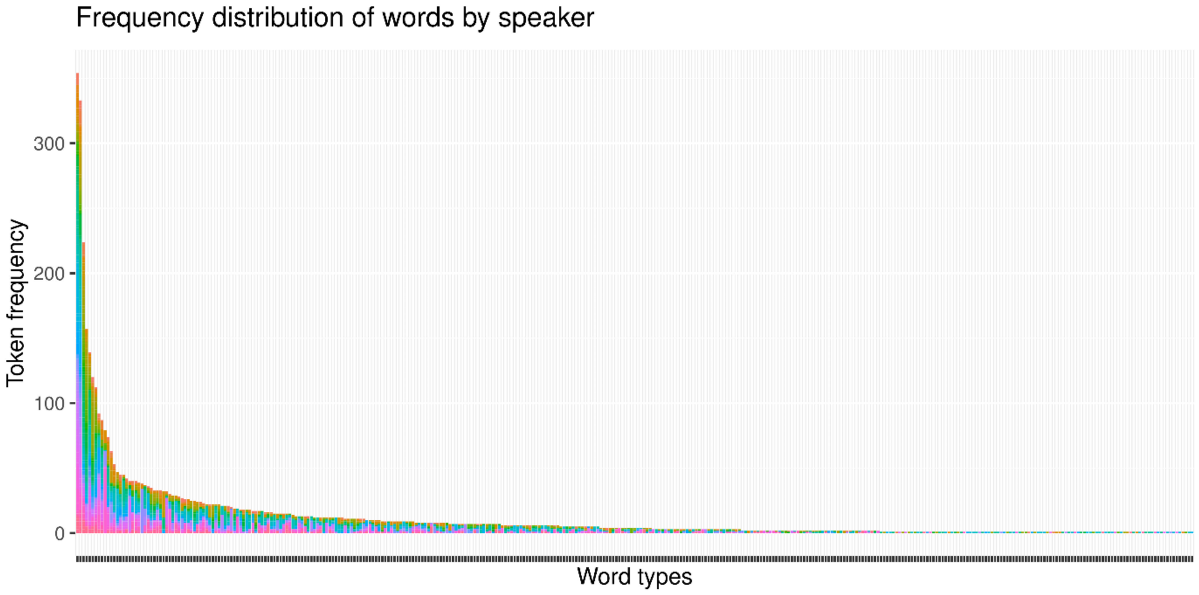


Supplementary Figure 8: Token frequency by word type and stacked by speaker. Different colors represent different speakers.

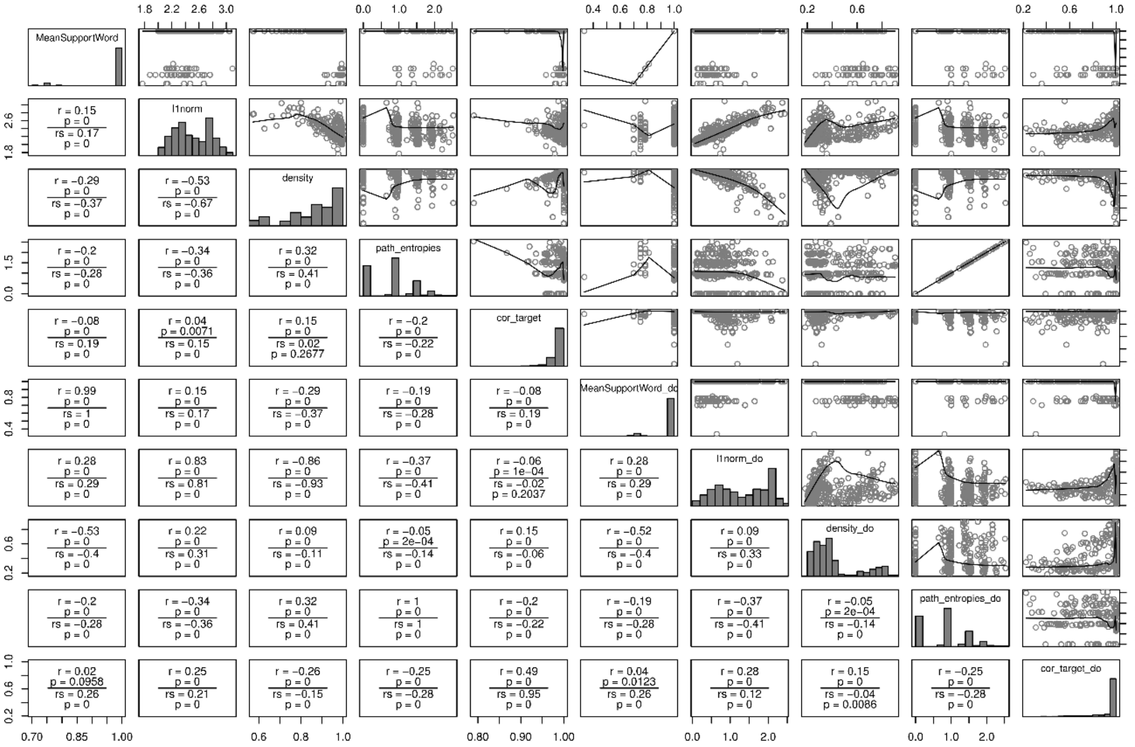

Supplementary Figure 9: Correlation matrix of LDL-derived variables in the Morphology Network and the Idiosyncratic Network (variables from the Idiosyncratic Network are marked with the ending _do).

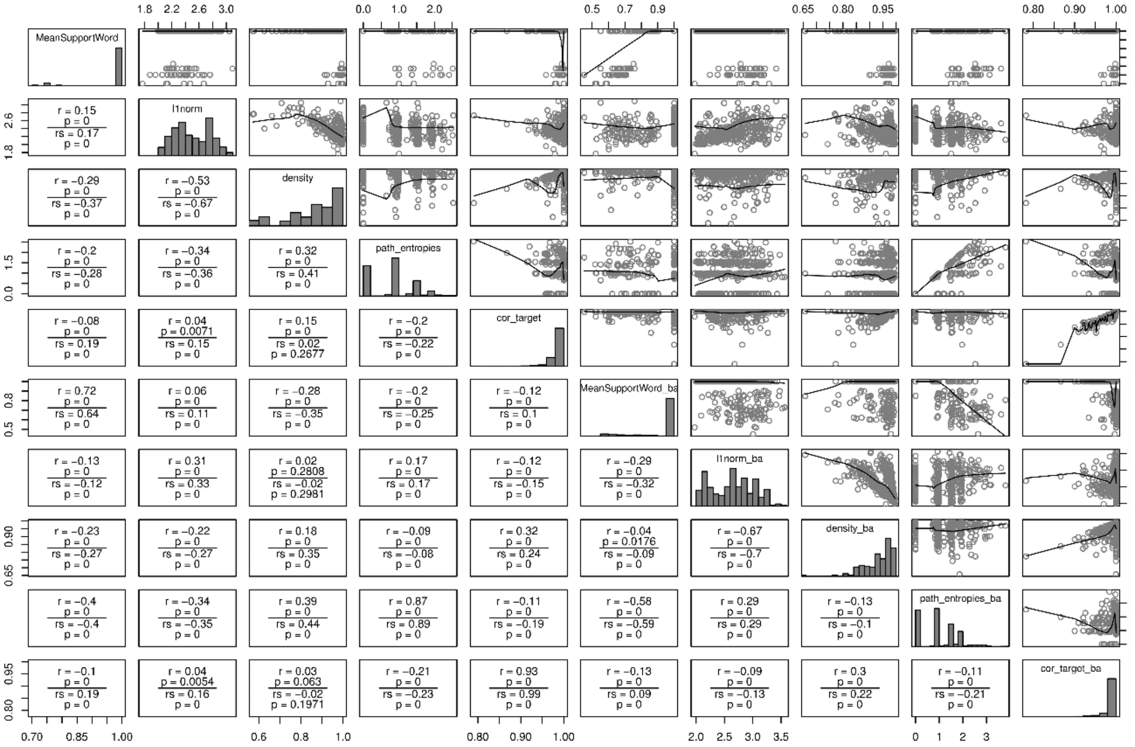

Supplementary Figure 10: Correlation matrix of LDL-derived variables in the Morphology Network and the Base Network (variables from the Base Network are marked with the ending _ba).

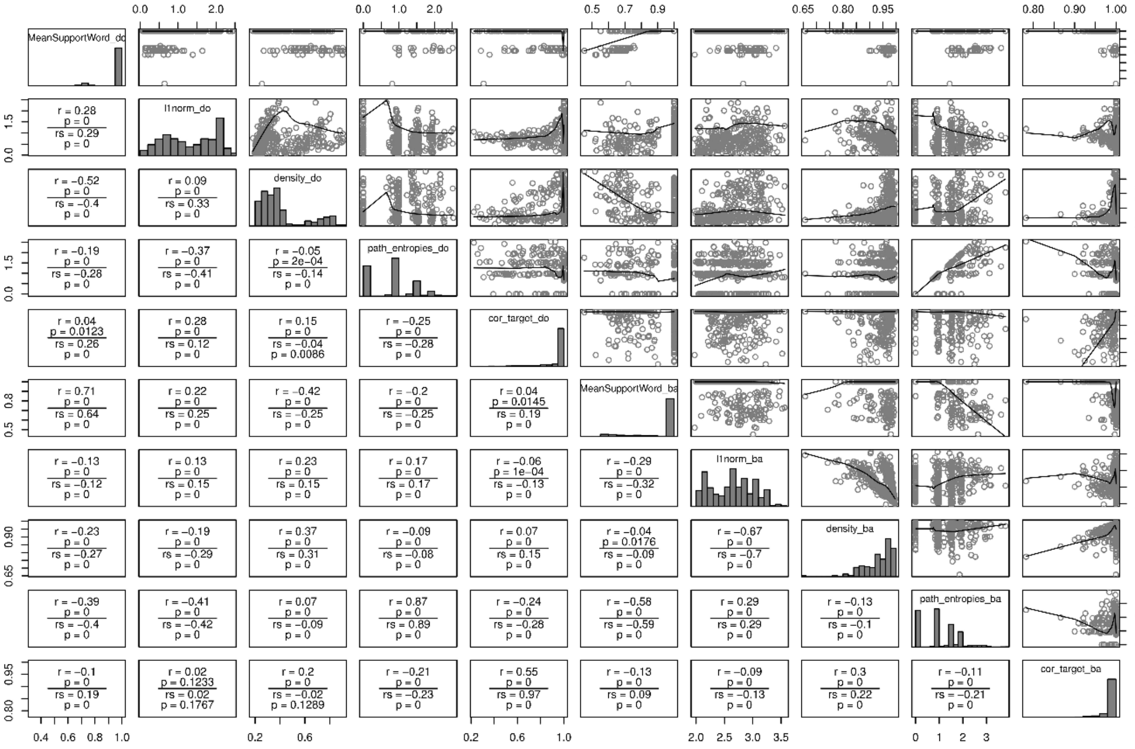


Supplementary Figure 11: Correlation matrix of LDL-derived variables in the Idiosyncratic Network and the Base Network (variables from the Idiosyncratic Network are marked with the ending _do, variables from the Base Network are marked with the ending _ba).

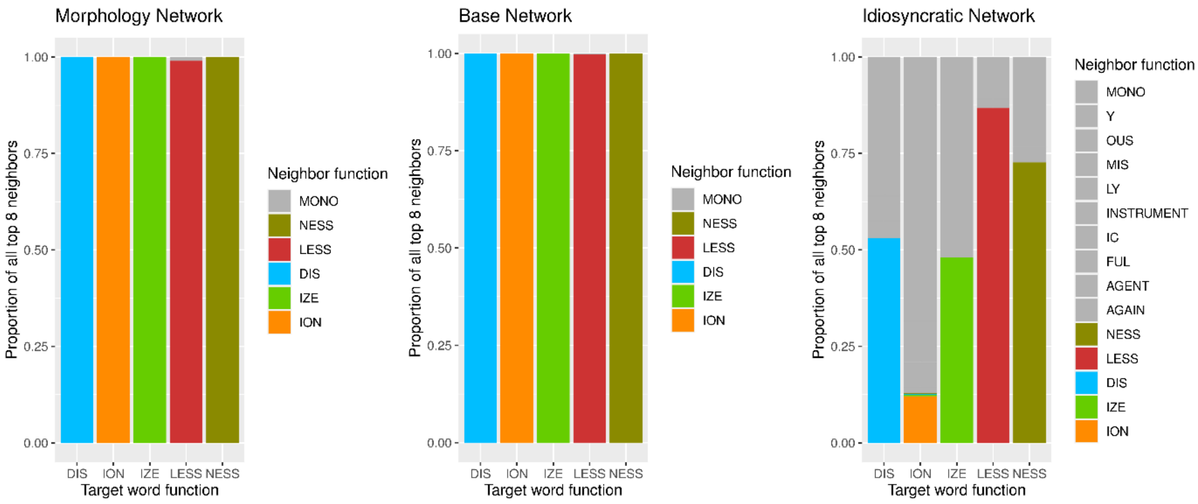


Supplementary Figure 12: Proportion of the morphological functions of the top 8 neighbors corresponding to the morphological functions of the target words in the three networks.
